# An Update on the Implications of New Psychoactive Substances in Public Health

**DOI:** 10.3390/ijerph19084869

**Published:** 2022-04-17

**Authors:** Ana Y. Simão, Mónica Antunes, Emanuel Cabral, Patrik Oliveira, Luana M. Rosendo, Ana Teresa Brinca, Estefânia Alves, Hernâni Marques, Tiago Rosado, Luís A. Passarinha, Maristela Andraus, Mário Barroso, Eugenia Gallardo

**Affiliations:** 1 Centro de Investigação em Ciências da Saúde (CICS-UBI), Universidade da Beira Interior, 6200-506 Covilha, Portugal; anaaysa95@gmail.com (A.Y.S.); antunes.ss.monica@gmail.com (M.A.); emanuel.cabral@ubi.pt (E.C.); patrik_99@live.com.pt (P.O.); may.rosendo@ubi.pt (L.M.R.); anabrinca99@gmail.com (A.T.B.); estefania.raquel5@gmail.com (E.A.); hefm976@gmail.com (H.M.); tiago.rosado@ubi.pt (T.R.); 2 Laboratório de Fármaco-Toxicologia, UBIMedical, Universidade da Beira Interior, 6200-284 Covilha, Portugal; 3 Serviço de Química e Toxicologia Forenses, Instituto Nacional de Medicina Legal e Ciências Forenses, Delegação do Sul, 1150-219 Lisboa, Portugal; 4 UCIBIO—Applied Molecular Biosciences Unit, Departamento de Química, NOVA School of Science and Technology, Universidade NOVA, 2829-516 Caparica, Portugal; 5 Associate Laboratory i4HB-Institute for Health and Bioeconomy, NOVA School of Science and Technology, Universidade NOVA, 2819-516 Caparica, Portugal; 6 Chromatox/Dasa Laboratory Ltda. Sumaré, São Paulo 01259-000, Brazil

**Keywords:** new psychoactive substances, health problems, toxicity

## Abstract

The emergence of new psychoactive substances has earned a great deal of attention, and several reports of acute poisoning and deaths have been issued involving, for instance, synthetic opiates. In recent years, there have been profound alterations in the legislation concerning consumption, marketing, and synthesis of these compounds; rapid alert systems have also been subject to changes, and new substances and new markets, mainly through the internet, have appeared. Their effects and how they originate in consumers are still mostly unknown, primarily in what concerns chronic toxicity. This review intends to provide a detailed description of these substances from the point of view of consumption, toxicokinetics, and health consequences, including case reports on intoxications in order to help researchers and public health agents working daily in this area.

## 1. Brief Introduction

According to the United Nations Office on Drugs and Crime (UNODC), new psychoactive substances (NPS) are classified as “substances of abuse, either in a pure form or a preparation, that are not controlled by the 1961 Single Convention on Narcotic Drugs or the 1971 Convention on Psychotropic Substances, but which may pose a public health threat”. Essentially the term “new” is used to report that a certain substance has emerged on the market illegally and is being used recreationally rather than medically [1]. Moreover, these substances can also be called “designer drugs”, since they are designed to mimic the effects of classical illicit substances that follow strict regulations from governmental entities and that are scheduled under drug control conventions [2]. In fact, the global occurrence of NPS poses major concerns and challenges for law enforcement agencies, but also for public health, due to constant diversification and growth. The more chemical and structural modifications are performed in a molecule, the more difficult it is for drug control legislations to track down these substances. To aggravate matters, NPS are often produced in clandestine laboratories, placing users at risk, since the purity and composition of such products is often unknown [3]. Hence, NPS continue to evade domestic and international laws and increase their availability on illegal markets [4].

A UNODC report from 2020 [5] showed more than 900 substances were documented in the 2009–2018 period, 65% of which were stimulants (phenethylamines and synthetic cathinones) and synthetic cannabinoid receptor agonists, with percentages of 35% and 30%, respectively, whereas opioids accounted for only 9% of NPS and 3% for sedative/hypnotics and dissociative substances, each. However, these numbers tend to change, and around 34% of synthetic opioids accounted for the most reported substances since 2018 [5].

Aiming at protecting public health, many Member States and the international community have developed a number of legislative approaches to efficiently combat the dynamics of the NPS market, namely concerning the rapid onset of the compounds, the manufacturers’ actions in order to elude control, and the lack of data allowing the evaluation of the extent of health damage. Therefore, several countries have adopted new legal measures to address this phenomenon, based either on existing laws focusing on consumers’ or health protection and promotion, or on developing innovative new legislation. In 2014, the Court of Justice of the European Union ruled that a substance could not be considered a medicinal product unless it had beneficial effects on human health, thus restricting the use of such laws for NPS control. However, not all countries share this approach, and a few have included NPS in internal laws concerning drug control policies or have passed legislation to control NPS considering their effect. Notwithstanding, institutions such as the European Monitoring Centre for Drugs and Drug Addiction (EMCDDA), the World Health Organization (WHO), and UNODC were called to explore creative solutions to address the problems related to the prosecution of non-controlled NPS.

The aim of this review is to present the classes of NPS that exist on the market, the main compounds within each family, how they are consumed, and statistics of consumption, toxicokinetics, observed toxic effects, and the consequences for health, including examples of case reports. Our objective is to describe these substances in detail in order to assist researchers and public health agents who work in this area on a daily basis.

## 2. Classification

As stated, NPS aim to imitate the classical and controlled drugs, such as cannabis, cocaine, heroin, lysergic acid diethylamide (LSD), or methamphetamine. For this reason, they can be divided and classified into different categories or categorized according to their psychotropic effects or nature [6]. For this review, we followed the same approach of the UNODC Early Warning Advisory (EWA) on NPS [6].

Some substances are used as stimulants, in this case, synthetic stimulants include aminoindanes, phenethylamines, piperazines, synthetic cathinones, and tryptamines, of which synthetic cannabinoids and synthetic cathinones are the largest groups [7,8,9]. As stimulant drugs, their biological action occurs mainly on the dopamine and serotonin neurotransmitters, and to a lesser extent on epinephrine, which is later responsible for the stimulatory effects of these substances [10]. 

Frequently sold as ecstasy, piperazines and derivatives are synthetic stimulants [11,12], that can be sold in night-out contexts among young people and are also available online [13]. 

In the aminoindanes group, chemical modifications (substitution of aromatic rings or *N-*alkylation, for example) occur on the amphetamine molecule, and substances such as 2-aminoindane (2-AI) can be produced. However, these substances are relatively new NPS, being originally produced and investigated due to their vasoactive and broncho dilating properties, as well as their analgesic properties [12,14].

Synthetic cathinones are chemically analogous to methamphetamines [8]. Substances as cathinones, amfepramones, mephedrone, 3,4-methylenedioxypyrovalerone (MDPV), and pentedrone are included within this class [8,12,15].

Another group of substances is phenethylamines [11]. These NPS include compounds similar in structure to amphetamines, where two phenethylamines form the “2-C series”, and a ring substitution forms the “D series” compounds. The phenethylamine core can be modified in eight different sites, originating as analogous derivatives [10].

Psychoactive tryptamines and derivatives are also considered NPS. They can be found in fungi, plants, and animals. An example of a NPS tryptamine is *N*,*N*-dimethyltryptamine (DMT). Other indolealkylamine molecules are also included in this category. Besides their stimulant effects, hallucinations are often experienced [12,16]. 

Synthetic cannabinoids mimic the effects of ∆^9^-tetrahydrocannabinol (THC), but they are reported to have a higher potency [17]. Different groups exist within synthetic cannabinoids, and substances are categorized therein based on their chemical structure. 

Phencyclidine (PCP)-type substances not only act as stimulants, but also as dissociative substances, that is, the main action of these substances affects the *N*-methyl-*D*-aspartate (NMDA) receptor in the brain, which ultimately leads to a dissociation from self and surroundings [18]. 

The UNODC classification also includes substances that are not included in the above-mentioned groups, either because they are structurally different or simply do not fit the categories. In the “other substances” category, substances that mimic the effects of classic hallucinogens (psychedelics) can be included [19]. Nonetheless, substances such as 4-bromo-2,5-dimethoxyphenylethanamine (2C-B) or DMT that could be in this category belong to the phenethylamines and tryptamines classes, respectively. We would like to highlight a subgroup in which synthetic depressants are included, synthetic opioids, whose main effect occurs by binding to opioid receptors, and are able to produce the same effects as classic opioids [20]. In addition, EMCDDA also includes new benzodiazepines or benzodiazepines analogues as NPS, with a core structure consisting of a benzodiazepine. In the beginning of 2021, close to 30 of these substances were being monitored in Europe, still only 4% accounted for the total seized NPS in Europe between 2009 and 2019 [21].

Moreover, khat (*Catha edulis*), kratom (*Mitragyna speciosa* Korth) and *Salvia divinorum* are all plant-based NPS [22]. Overall, these substances are consumed in the form of fresh leaves, but dried leaves are also available. Due to their nature, these substances are generally chewed, smoked, infused, or brewed for consumption as infusions [12]. Regardless, powder formulations, seeds, or liquid extracts are also available [12,22,23]. Such substances were originally intended to be used in traditional medicine to treat various symptoms such as pain, fever, or even diarrhea [22,24]. Besides the UNODC classification, other plant-based substances exist, as is the case of ayahuasca, for which psychoactive effects are due to the presence of a tryptamine called *N*,*N*-dimethyltryptamine (DMT), which falls in the tryptamines category [16,25]. Psilocybin, the main constituent of “magic mushrooms” also belongs to the tryptamines category, according to the UNODC, due to its hallucinogenic effects exerted on serotoninergic neurons [22]. Other plant-based psychoactive substances are present in “iboga”, “mandrake”, and “kava”, for example, but there are others, which are not classified as NPS [22]. 

Some of these substances begin to be more familiar to researchers; still it is sometimes difficult to find comprehensive studies about their toxicity to humans, whereas the available information is based mostly on animal research or fatal poisoning cases. The majority of NPS have very little or no history at all about medical use, which is fundamental to study long-term exposure and evaluate acute toxicity and long-term risks. In recent years, excellent reviews have been published on the problem of NPS consumption; however, these reviews have only included some NPS groups [7,22,24,26,27,28] focused on the public health issue [29] as well as the social, political, and economical aspects of NPS markets [30]. However, these latter reviews [29,30] do not delve deep into the subject, namely the different classes of NPS on the market, pharmacokinetics and pharmacodynamics, as well as toxicological aspects. Throughout this review, more detailed information can be found about each group, regarding chemical structures, toxic effects and pharmacokinetics, consumption statistics, and other key findings. This review intends to fulfill this gap with all updated information compiled.

Three electronic databases were used for the systematic literature search: Medline, ISI Web of knowledge, and Google scholar. Search strings were “piperazines”, “piperazines derivatives”, “benzylpiperazine derivatives”, “phenylpiperazine derivatives”, “piperazines characteristics”, piperazines cases of abuse”, “aminoindanes”, “phenethylamines”, “synthetic cathinones”, “synthetic cannabinoids”, “arylcyclohexylamines”, “phencyclidine and or analogues”, “ketamine”, “MXE”, “4-MeO-PCP”, “3-MeO-PCP”, “tryptamines”, “psychoactive substances of natural origin”, “NPS” and (or) “pharmacokinetics”, “therapeutic properties”, “toxicology”, and “effects”, and “case report” (all fields), and only papers from 2006 to present were selected. However, concerning aminoindanes, phenethylamines, synthetic cathinones, and “psychoactive substances of natural origin”, only the last 10 years were included due to the high number of papers available. The articles were independently selected by three of the authors for each class of NPS to determine their relevance in the context of the current review; only articles that were selected by at least two of the authors were included.

Furthermore, several articles published since the 1960s were also relevant considering that khat and tryptamines have been intensively studied for decades. Information in books was also important, especially concerning general aspects.

### 2.1. Piperazines

**Figure 1:**
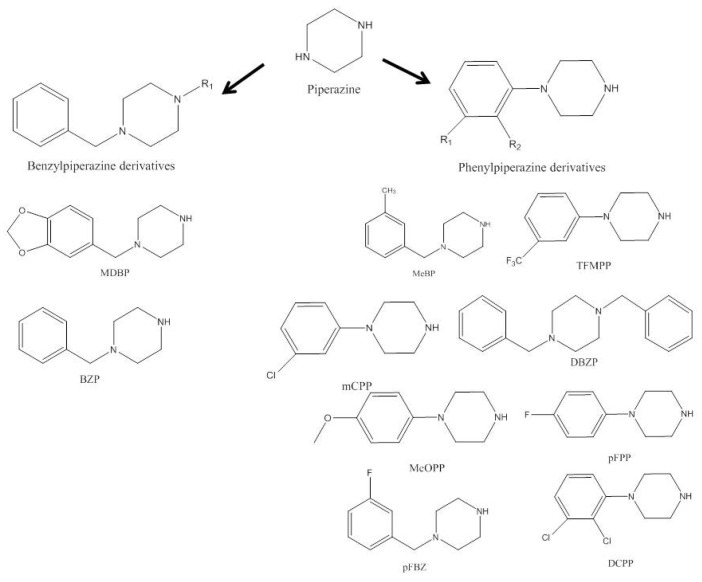
Represents the chemical structure of some of piperazines.

Piperazines were the first drugs of abuse deriving from phenylethylamine-based compounds. They are completely synthetic and have been widely sold as legal party pills, powder of pure substances, and adulterants due to their stimulant pharmacological properties. Piperazine (Figure 1) is an *N*-heterocyclic bioactive natural product extensively present in biologically active compounds [13,31,32,33], and was used as anti-helminthic [34]. 

The two main structural groups that form the piperazine-based compounds are 1-benzylpiperazine and 1-phenylpiperazine (Supplementary Material Table S1 and Figure 1) [10,13,31,32,35,36,37,38,39,40].

In the second half of the 1990s, piperazine designer drugs emerged in the illicit market, but the United States and Scandinavia were the only regions reporting high consumption rates [31,35,41]. *N*-benzylpiperazine (BZP) was the first consumed derivative of piperazines in 1996 in the United States. It is usually combined in pills with 1-(3-trifluoromethylphenyl)piperazine (TFMPP), giving rise to the much sought-after 3,4-methylenedioxymethamphetamine (MDMA) in humans, despite being weaker in comparison [13,35,36]. The appearance of new piperazines in Europe has been irregular, and the first case of piperazine abuse reported in Europe occurred in Sweden in 1999. These were not reported until 2006 by the EU Early Warning System (EWS). Subsequently to their first identification, piperazines appeared and were detected from 2005 to 2019. Only in 2004 they were considered NPS, and the EU proceeded to control their consumption in 2008 [42]. In England and Wales, between 2008 and 2009, deaths caused by drugs of abuse mainly involved piperazines. Currently, these compounds have spread worldwide, and around 10% of the population from developed countries has already used these types of designer drugs [31,35]. Piperazines are among the leading deaths caused by synthetic psychoactive stimulants and hallucinogens [43]. 

Piperazine derived drugs can be consumed as capsules, tablets, loose powders, or in liquid forms, and can also appear as adulterants of MDMA and cocaine [13,31,35]. Therefore, they can enter the system through oral ingestion, nasal insufflation, injection, and smoking [35].

Doses can vary between 50 and 250 mg [32,35]. Party pills contain 28 to 133 mg of BZP, with an average level of 65 mg per tablet, even though some reports have presented this amount to be up to 1000 mg. Party tablets show a composition of 2:1 to 10:1 of BZP and TFMPP, respectively. (1-(3-chlorophenyl)piperazine) (mCPP) rounded 45 mg per pill [44,45,46,47]. These compounds are rapidly absorbed from the gastrointestinal tract, subsequently metabolized by the liver, and finally excreted in the urine in their conjugated form. Benzyl piperazines are more stable and barely metabolized as opposed to phenyl piperazines. The latter are extensively metabolized and excreted as metabolites, making it more difficult to find the parent compound in human samples [13,31,36,37]. They are also redistributed by different organs, such as the kidney, liver, and brain [36]. It has been proposed that these compounds are metabolized in the liver by CYP2D6, CYP1A2, and CYP3A4, isoenzymes belonging to the cytochrome P450 superfamily [35,36]. Despite these findings, recent studies have determined high concentrations of BZP, TFMPP, and mCPP in body fluids, such as blood, 24 h urine and 30 h plasma samples after the ingestion of a standard portion of 200 mg. Consequently, in overdose cases, the original drug should be detectable, even though the results might be discrepant between different subjects [31,35,37]. 

Anita et al. determined that after consuming an average dose of BZP (200 mg) this drug showed an elimination half-life (t1/2) of 5.5 h and clearance of 99 L/h. TFMPP exhibited two half-life disposition phases, 2.04 h (±0.19 h) and 5.95 h (±1.63 h) respectively, and a clearance of 384 L/hour (±45 L/h). In turn, mCPP presented a high variability clearance (11–92 mL/h) and bioavailability (14–108%) [35,48,49,50]. Hondebrink et al. estimated that the concentration ranges of BZP, TFMPP, and mCPP in the brain were 0.2–36 μmol/L, 22–89 μmol/L, and 1.7–85 μmol/L, respectively [49].

Piperazine derivatives increase the levels of dopamine, serotonin, and noradrenaline [32,51] by influencing their reuptake transporters (DAT, SERT, and NET, respectively) [32,52]. These compounds are less potent than amphetamines, MDMA, and methamphetamine [31,35].

BZP is the most studied compound [13,32,53], causing motion disorders, tremors, hypertension, urinary retention, vomiting, and paranoid psychosis related to dopaminergic activity, while anxiety, insomnia, dilated pupils, dizziness, palpitations, and chest pain are due to its adrenergic properties [13,32,54]. Cholinergic effects include vomiting, xerostomia, immunological disorders, hypersensitivity reactions, and pruritus [32,54]. Lastly, serotonergic activity results in agitation, dissociation states, nausea, confusion, abdominal pain, and headaches [32]. The dopaminergic effects of BZP are the increase in positive agitation, and the reaction to uncertainty decreases. TFMPP increases the emotional response [32,55].

The combination of BZP with mCPP or TFMPP can mimic ecstasy’s characteristics, where TFMPP and mCPP act as serotoninergic agonists and BZP releases DA [32,56]. In psychiatric practice, mCPP is used to study serotonin function showing dose-dependent effects. Piperazine derivatives also present toxic effects on cardiac cells, since they disturb Ca^2+^ homeostasis, provoke adenosine triphosphate (ATP) depletion and loss of mitochondrial membrane potential, leading to cell death. Disruption of Ca^2+^ homeostasis can alter heart rate [13,31,32]. 

Besides the previously mentioned effects, piperazines can cause psychiatric disorders, such as insomnia, drowsiness, fatigue, lowered mood, tension, depression, euphoria/dysphoria, mood swings, panic, hallucinations, and withdrawal from social interactions [13,32,51,52,54,57,58,59,60,61]. They also cause cardiovascular effects, such as increased systolic and diastolic blood pressure, tachycardia, palpitations, myocardial infarction, circulatory collapse, and prolongation of the QT interval [32,52,54,57,58,60,61,62,63,64,65]. At the neurological level, they cause, dizziness, blurred vision, convulsions, epileptic seizures, increased sensitivity towards light and noise, and poor concentration [13,32,52,54,57,58,60,61,62,66]. They cause metabolic and respiratory acidosis, hyponatremia, increases in plasma prolactin, adrenocorticotropic hormone, and cortisol [13,32,52,54,58,61,62]. Dehydration and lack of appetite are symptoms at the gastrointestinal level [13,32,52,54,57,59,60,61,62,67,68]. Piperazines also affect the kidney, and initiate renal failure, acute kidney injury, and affect lungs, causing hyperventilation [13,32,52,54,58,61,62]. At the muscle level, the symptoms are rhabdomyolysis and muscle contractions [32,52,54,60]. Piperazines still have other complications such as hyperthermia, abundant sweating, bruxism, and chills, and can affect the skin [13,32,52,54,57,60,61,62]. The heaviest complications are multiple organ failure, hepatotoxicity, intravascular coagulation, serotonin syndrome, attempted suicide, nephrotoxicity requiring dialysis, malignant neoplasms, autoimmune diseases, and can lead to death [13,32,45,51,52,54,57,58,62,69,70,71,72].

There is a broad age range for consumption, with young adults being the most significant consumers. There are many fatal and non-fatal reports, but some cases may have gone unnoticed since piperazines are usually not targeted in hospital toxicological analysis [35]. Several non-fatal cases are shown in Supplementary Material Table S2. 

### 2.2. Aminoindanes 

Aminoindanes have been investigated as research chemicals. They can act in the dilation of blood vessels, enabling strong potential for aiding in respiratory processes and thus leading to acting as strong bronchodilators. The first development regarding their research relied on these properties [73,74,75]. 

In 1944, an animal study conducted by Levin et al. demonstrated that 2-AI and other derivatives were more effective vasodilators than *L*-ephedrine, but without showing the toxicity of other amphetamines [76]. In 1961 and 1963, two different research papers investigated the analgesic potential of these compounds [77,78]. In 1973, aminoindanes were also investigated in the context of neurodegenerative diseases. Martin et al. conducted an animal study in which a few aminoindanes derivatives were tested for anti-Parkinson effects, but no evidence that they could reduce or even act on the disease’s symptoms was observed in spite of monoamine oxidase (MAO) being inhibited by some compounds [79]. The MAO-B inhibition allows for a longer action of dopamine in the brain, ameliorating Parkinson’s symptoms [80]. Martin et al. concluded that the biological effect of aminoindanes is directly related to the position and type of the amine substitute [81].

**Figure 2:**
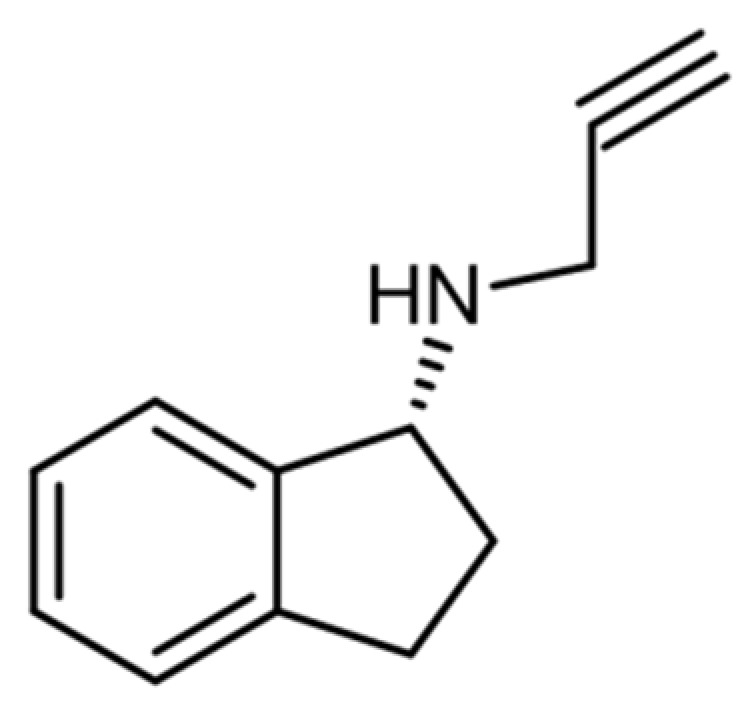
Chemical structure of rasagiline.

The inhibitory effect of aminoindanes on MAO-B was further investigated by Kalir et al. in 1981, leading to the identification of a strong inhibitor that did not reveal any cardiovascular or central nervous system (CNS) adverse effects. This compound was rasagiline (Figure 2), which was used in the treatment of Parkinson’s disease [82].

Selegiline is an analog of rasagiline, its main metabolites are L-amphetamine and L-methamphetamine, whereas rasagiline does not metabolize into these compounds [82,83].

**Figure 3:**
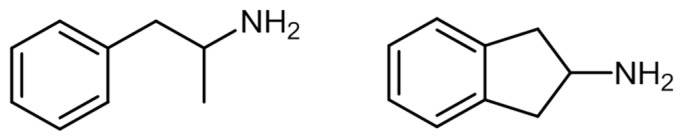
Chemical structure of amphetamine (**left**) and 2-AI (**right**).

The chemical structure of aminoindanes is very similar to that of amphetamines (Figure 3) [14].

In 1986, Nichols et al. defined a group of psychoactive substances for therapeutic use featuring molecules such as MDMA, 3,4-Methylenedioxyamphetamine (MDA), and 5,6-methylenedioxy-2-aminoindane (MDAI) [84,85]. The authors conducted a study using an animal model to understand the differences between the use of MDMA and aminoindanes, such as MDAI. The results pointed out that aminoindanes mimic the effects of MDMA and other derivatives without neurotoxicity in 5-HT receptors [86]. In 2014, Simmler et al. [56] performed a monoamine transporters (MATs) inhibition test on human embryonic kidney cells and concluded that MDAI, 2-AI, and 5-iodo-2-aminoindane (5-IAI) had lower inhibitory effects than MDMA.

Around 2010, and due to their similarity to amphetamines, aminoindanes emerged in the United Kingdom’s illicit drug market as a new class of NPS-synthetic aminoindanes posing as alternatives to legislated compounds from other classes [87]. 

The ability of these compounds to mimic the effects of serotonin-releasing drugs, such as MDMA and other amphetamines, makes them effective alternatives to these drugs. Furthermore, these compounds stimulate the CNS, mediating the action of serotonin, norepinephrine, and dopamine [88]. MDMA arose recent interest for the treatment of post-traumatic stress disorder [89]. However, its application in other comorbid psychiatric conditions has been poorly investigated. Some studies have proven pro-cognitive effects, such as improving social connectedness and mood, increased responsiveness, and emotional sensitivity. However, other studies have also proven anti-cognitive effects [89]. 

**Figure 4:**
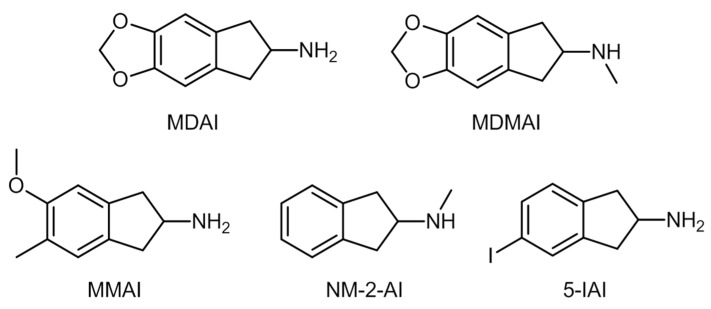
Chemical structures of synthetic aminoindanes.

These synthetic aminoindanes derive from 2-AI (Figure 4), some of the most common originating from structural modifications on the 2-AI precursor molecule [88]. Some examples of these molecules are MDAI, 5,6-Methylenedioxy-*N*-methyl-2-aminoindane (MDMAI), 5-methoxy-6-methyl-2,3-dihydro-1*H*-inden-2-amine (MMAI), *N*-methyl-2,3-dihydro-1*H*-inden-2-amine (NM-2-AI), and 5-IAI.

There is still a lack of general information about these substances, mainly concerning their pharmacological effects, which makes the legislation of these compounds challenging. The UNODC reports that none of these NPS is under international control [88,90]. In fact, until 2012, aminoindanes were the second smaller NPS group reported to the UNODC, with just 34 reported cases at a global scale against 684 of synthetic cathinones [12].

Synthetic aminoindanes are usually found as powders and crystals, and are consumed through ingestion, snorting, or rectal application. No reported cases of the injection or smoking of these substances exist [91,92,93]. Common street names are “*MDAI gold”* or “*Pink Champagnes”,* the former for MDAI-based substances and the latter for preparations containing 2-AI [94].

Information on the effects and consequences of the consumption of these compounds has been found in forums of users’ experiences, such as Erowid. Some known effects are stimulation, empathy, and euphoria, while adverse effects include anxiety, depression, and tachycardia [14]. Users have reported the onset of effects for these substances being about 30 min after oral consumption, and the window for maximum effect is between 45 min and 3 h after ingestion [95].

Manier et al. [75] investigated in vivo and in vitro metabolic pathways for 2-AI and NM-2-AI. The former is metabolized via *N*-acetylation, while the latter suffers hydroxylation. The authors identified the formation of hydroxylamine in the metabolic pathway of this last compound, which can be toxic for humans or animals at high concentrations [96].

Palenicek et al. in 2016 [97] demonstrated that MDAI (administered to rats at 10 mg/kg) was rapidly absorbed in the brain, even though the larger concentration was found in the lungs. The authors observed increased and selective neurotoxicity for this compound. Some studies on animal models stated that the intake of aminoindanes at larger doses increases neurotoxicity and body temperature, which can originate toxic conditions (for instance, serotoninergic syndrome) [14,88].

Aminoindanes act primarily on 5-HT-receptors, and therefore stimulation is not as high as occurs when other compounds (cocaine, MDMA) are used. Thus, users consume higher doses of the compounds or mix them with other stimulant drugs, which has a potential risk of cardio- and neurotoxic conditions [91,98].

Gallagher et al. reported in 2012 that MDAI has been linked to several toxic conditions, such as renal and hepatic failure, valvular heart disease, and respiratory distress syndrome [99]. *Postmortem* situations related to MDAI, 5-Iodo-2-aminoindane (5-IAI), and 2-AI have been reported [91,100], the most illustrative example being a young woman who died of cardiac arrest, showing MDAI concentrations of 26.3 mg/L [91].

### 2.3. Synthetic Cathinones

Cathinone occurs naturally in the leaves of khat [101], a plant usually found in East and Southern Africa, Southwest Arabian Peninsula, and Afghanistan [102,103]. More details on khat are presented in the plant-based NPS section.

**Figure 5:**
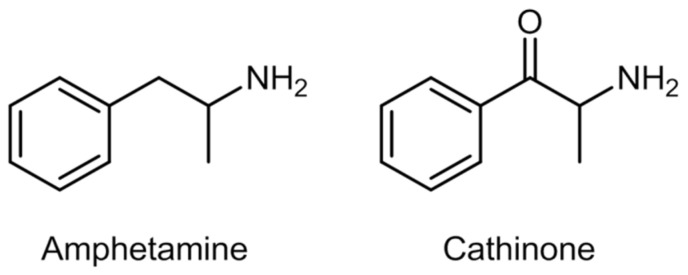
Chemical structure of amphetamine and cathinone.

In 1975, cathinone, or 2-amino-1-phenylpropan-1-one, was identified and isolated from fresh khat leaves [101]. This natural compound is a β-keto analogue of amphetamine, hence its amphetamine-like stimulant effects [101]. Figure 5 shows the chemical structure of amphetamine and cathinone.

However, synthetic cathinones were first synthesized a few decades before, starting with methcathinone in 1928 [104], and mephedrone one year later [105]. The structural resemblance of these compounds with classical amphetamines and their effects on the CNS brought the use of these compounds for clinical purposes to attention [101,106,107], primarily as antidepressant and appetite suppressant drugs [108,109,110]. It is also relevant to point out that only one synthetic cathinone is still available on the market for therapeutic purposes, bupropion, which is used as an antidepressant, coadjutant in smoking cessation therapy, and in the treatment of obesity [101,111,112].

**Figure 6:**
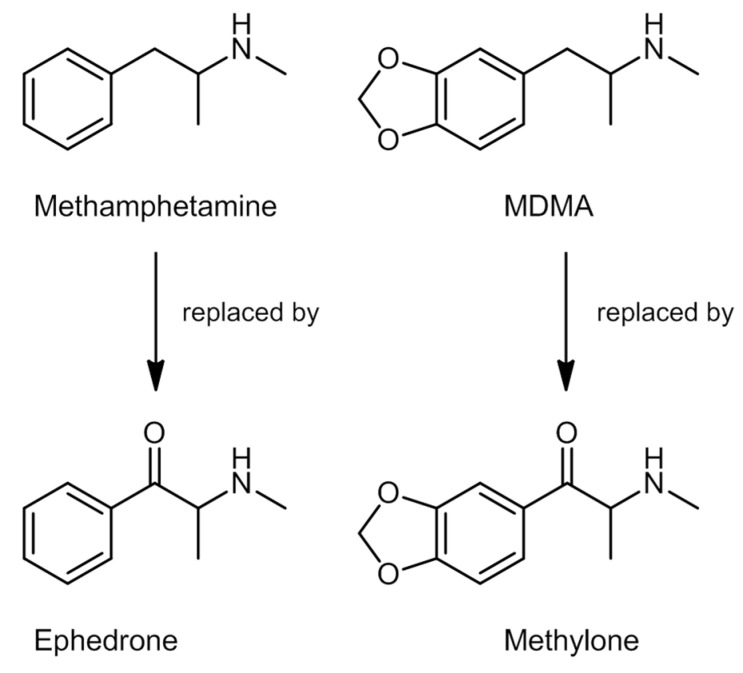
Chemical structures of methamphetamine, MDMA, ephedrone, and methylone.

Despite their medicinal use, access to these compounds has been limited, considering their potential for abuse and dependence, and the adverse effects they might cause [113]. Methcathinone was the first compound used recreationally in the Soviet Union in the 1970s. Synthetic cathinones are commonly known as amphetamine derivatives not only because of their similar chemical structure to amphetamine (Figure 5), but also concerning their physiological and behavioral effects [101,114]. That said, in the beginning of the 21st century, specific cathinones started to appear in the market as legal substitutes for illegal substances, such as methamphetamine and MDMA [114], that were replaced by ephedrone and methylone-1-(1,3-benzodioxol-5-yl)-2-(methylamino)propan-1-one, respectively (Figure 6).

**Figure 7:**
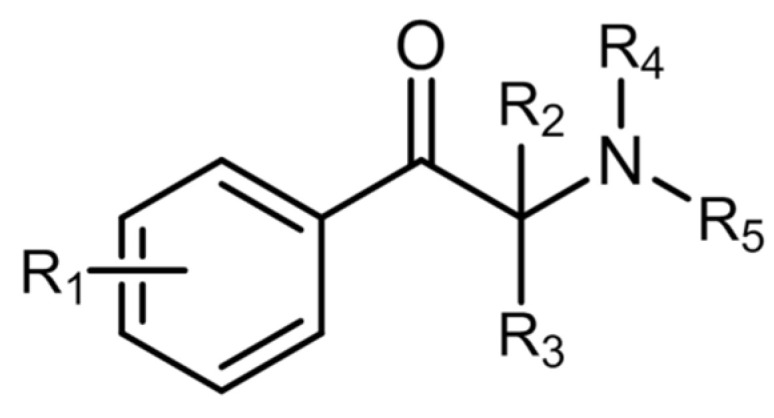
General chemical structure of synthetic cathinones.

Innumerable cathinone derivatives can be synthesized by modifying the cathinone backbone structure. There are five different sites in which these modifications can occur (Figure 7) [101,114]. A total of 156 synthetic cathinones are currently monitored by the EU EWS, making it the second largest group of substances controlled by this organization [115]. 

These compounds have gained outstanding popularity worldwide [101] due to their wide availability, relatively low prices, impressive marketing strategies, high purity compared to “street drugs”, and popularization via the internet and in the so called smartshops. They are also known by the street names “Miaow Miaow”, “M-Cat”, “Msmack”, “Drone”, “Fert”, or “Bubbles” [116]. Regulatory measures have been introduced to control these substances. However, new uncontrolled derivatives keep appearing on the market to replace those that have been subjected to legislation [101]. 

Some cathinone derivatives are internationally controlled. Cathinone and methcathinone are listed in Schedule I of the 1971 Single Convention on Psychotropic Substances, cathine in Schedule III, and pyrovalerone in Schedule IV [8].

Methylone was the first synthetic cathinone reported to the EMCDDA in 2005, and it is part of the first generation of synthetic cathinones to be marketed as “legal high” [8,101]. The legislative control of the first generation of synthetic cathinones and the demand for novel and legal substitutes increased the synthesis of new molecules. 

**Figure 8:**
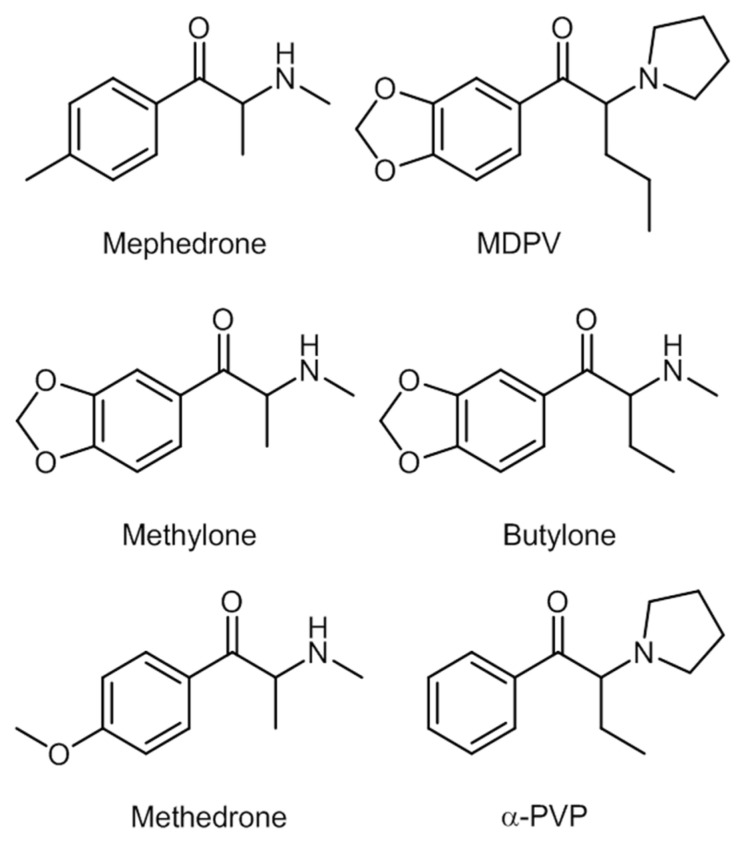
Some of the most common synthetic cathinones.

Mephedrone and MDPV appeared two years after methylone. Other compounds appeared ever since, for instance, the commonly found butylone, 4-methylethcathinone (4-MEC), and methedrone; and the less common α-pyrrolidinopentiophenone (α-PVP) and buphedrone [8]. Some of these synthetic cathinones are shown in Figure 8.

Some cathinone derivatives show only about half the stimulant potency of amphetamines [117], and this leads to the consumption of higher doses or even re-dosing [114]. 

These NPS are mainly consumed orally [118,119], but nasal insufflation and inhalation, gingival and sublingual mucosal routes, intravenous, intramuscular, and subcutaneous injections [120,121], and rectal administration [8] may be used as well. 

Cathinones can be consumed pure or in combination with another cathinone and/or with various other types of NPS, illicit drugs, medicines, anesthetic agents, and alcohol, to enhance the psychoactive effect [118]. Concomitantly used with prescription drugs, some side effects can be reduced: BZPs help with anxiety; β-blockers are used with tachycardia; proton pump inhibitors help with stomach pain; and selective phosphodiesterase type 5 inhibitors increase libido and improve sexual performance [119,122]. However, this concomitant consumption may be unintentional if the information about the content of drug is lacking.

Doses vary largely from a few milligrams to a couple of grams depending on the substance that is being consumed. The route of administration is also important: when a synthetic cathinone is consumed by nasal insufflation, there is a quicker onset of the desired effects, so lower doses are needed in comparison to oral ingestion [118]. A typical dose of mephedrone, for example, is 100–250 mg. On the other hand, MDPV is usually consumed in doses of 5–10 mg because it is more potent [123]. 

In the United Kingdom, toxicity related to synthetic cathinones consumption increased from none to 600 cases from 2009 to 2010. Additionally in that year, the prevalence of consumption in Northern Ireland was about 2%. In France, MDPV and 4-Methylethcathinone (4-MEC) were two of the most consumed NPS.

After consumption, these substances are first distributed to heavily irrigated organs such as brain, lungs, liver, and kidneys. It is in this phase that the first effects occur [101]. Synthetic cathinones show the ability to mediate the activity of monoamine transporters and receptors in the brain. These substances interact mainly with the serotonin receptor and transporters such as DAT, NET, and SERT. Synthetic cathinones block these receptors and transporters which, in turn, leads to an accumulation of monoamines such as dopamine, serotonin and noradrenaline in the synaptic cleft [56,101,124,125,126,127,128]. Afterwards, distribution occurs throughout muscle, fat, and skin accounting for the redistribution of some drugs. Several factors condition this process, such as the ability of the substance to bind to plasma proteins and tissues, cardiac output, blood flow and capillary permeability, and local pH. In general, synthetic cathinones tend to bind poorly to plasma proteins [101].

Synthetic cathinones are subjected to phase I and II metabolism, mostly mediated through cytochrome P450. Notwithstanding, cathinones are also found non-metabolized in urine [101,118,129]. Chemical structure determines the main metabolic pathways for each substance [101]. According to the EMCDDA, there are still few studies regarding ring substituted cathinones, pharmacokinetics and pharmacodynamics. Although, some studies with methcathinone in rats and humans indicate that *N*-demethylation and hydroxylation of keto groups and oxidation of alkyl groups in the ring are common pathways [123]. Nevertheless, the fact that metabolism varies according to structures and that it is dependent on numerous other factors leads to an increased complexity, which results in a higher risk and unpredictable consequences when these substances are consumed simultaneously with others [56,101,124,125,126,127,128].

Average half-life times have been reported for some of these substances, for instance between 1.5 and 4.3 h for cathinone [130,131]. 

Several pharmacodynamics and pharmacokinetics studies have been conducted using animal models. Novellas et al. identified the elimination half-life time of MDPV to be around 1 h [132]. Even though in vivo studies in humans are less common, mephedrone was described by Papaseit et al. as having an average half-life of elimination of 2.15 h, following administration of 200 mg doses to humans. The maximum concentration in plasma was reached in 1.25 h, and this substance was undetectable after 24 h [133]. 

In phase I metabolism, the formation of hydroxyl metabolites is one of the most common and major metabolic pathways. *N*-dealkylation is the major metabolic pathway for cathinones presenting *N*-alkyl groups, whereas for methylenedioxyphenyl cathinones, some metabolites will result from demethylation followed by *O*-methylation. For *N*-pyrrolidine cathinone derivatives, most metabolites will result from hydroxylation followed by dehydrogenation. The resultant compound is then further metabolized originating an aliphatic aldehyde [134]. Glucuronidation is a common way of phase II metabolism. 

In 2015, Negreira et al. using microsomes of human liver concluded that methedrone is metabolized via *N*-demethylation, *O*-demethylation, and hydroxylation of the aliphatic side chain, as well as reduction of the β-ketone moiety [135]. Kamata et al. reported metabolic pathways for methylone in both humans and rats, in which the β-ketone moiety reduction was not observed. The authors reported that, although a percentage of the dose was excreted unchanged, processes of *N*-demethylation and demethylenation, followed by *O*-methylation occurred. The most common metabolites in urine were 3′-hydroxy-4′-methoxymethcathinone and 4′-hydroxy-3′-methoxymethcathinone. Židková et al. reported for the same compound the metabolites 3′-hydroxy-4′-methoxycathinone and 4′-hydroxy-3′-methoxycathinone in rat urine [136].

Concerning adverse effects, neurological, psychiatric, and cardiac systems are the most affected systems in the body. Other effects may also occur, which can lead to multiple organ failure and death [119,120,121,122].

The most common neurological and psychiatric effects include agitation and anxiety, cognitive disorders, visual and auditory hallucinations, delusions, aggressive and erratic conduct, paranoia, psychosis, and seizures. Cases of stroke, encephalopathy, coma, and convulsions have also been reported [137,138,139,140,141]. Concerning cardiac effects, chest pain, hypertension, tachycardia, and cardiac arrest are documented [101]. Other body compartments can be affected, namely the gastrointestinal and hepatic, hematological, and respiratory systems [119,120,121,122]. The repeated use of these substances at high doses may cause craving, dependence, tolerance, and withdrawal syndrome, and these effects have been documented [101,118,119,120].

There is little information concerning the pharmacology features and toxicity of these substances [142], which would be important to help clinicians dealing with intoxications. This is particularly relevant in acute intoxications, considering that the consumed substances are not known and several symptoms overlap with those induced by other situations or drugs of abuse (amphetamines, cocaine, and MDMA) [101]. 

Because these substances are synthesized and sold in illicit markets, abusers do not precisely know what they are ingesting, often leading to unwanted effects, overdoses, and death [129,142,143].

The first fatal case associated with the consumption of synthetic cathinones occurred in Sweden, in 2008, with mephedrone. Later, several other fatal cases concerning this substance appeared, although in most cases other drugs were also detected [8]. The same happens with other synthetic cathinones, since they are usually consumed in drug cocktails.

In 2019, Vignali et al. reported a fatality of a 27-year-old man, and 1-phenyl-2-(pyrrolidin-1-yl)hexan-1-one (α-PHP) was detected in several samples [144]. In 2020, Adamowicz et al. reported a fatality of an 18-year-old man, in which 4-methyl-1-phenyl-2-(pyrrolidin-1-yl)pentan-1-one (α-PiHP) and 1-(4-chlorophenyl)-2-(methylamino)-1-propanone (4-CMC) were present [145]. Lelièvre et al. also reported a fatality, in this case, a 39-year-old man, linked to the consumption of synthetic cathinones in 2020. Several of these compounds were detected in *postmortem* analysis, including 2-(ethylamino)-1-(4-methylphenyl)-1-pentanone (4-MEAP) and 1-(4-methylphenyl)-2-(1-pyrrolidinyl)-1-hexanone (MPHP) [146].

### 2.4. Phenylethylamines

**Figure 9:**
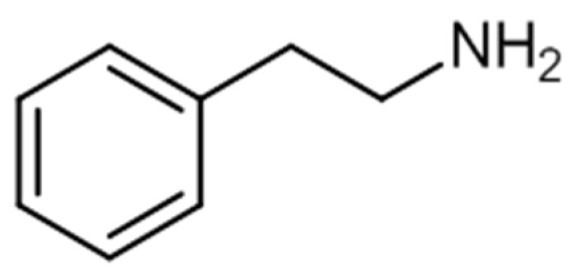
Chemical structure of phenylethylamine.

Phenylethylamines are derivative compounds of phenylethylamine (Figure 9), an organic compound and a natural monoamine alkaloid that acts as a stimulant in the CNS, and can act as an antimicrobial agent [147,148].

Phenylethylamines include many compounds capable of producing stimulant and psychoactive effects.

Some of the first phenylethylamines were synthesized early in the last century. 1-(4-methoxyphenyl)-*N*-methylpropan-2-amine, or P-methoxymethamphetamine (PMMA), for example, was first reported in 1938 [149]. Later, during the last two decades of the 20th century, other phenylethylamine derivatives were synthesized, featuring compounds from both 2C and D series. A major example is 2C-B, which derives from a natural phenylethylamine [150]. Furthermore, in 1997, the studies of David Nichols’ team reported that the hallucinogenic effects of compounds such as 2C-B or dimethoxybromoamphetamine (DOB) were more potent than other natural hallucinogenic compounds [151]. From that point on, several other phenylethylamines were synthesized, such as benzodifuranyl substances [152].

**Figure 10:**
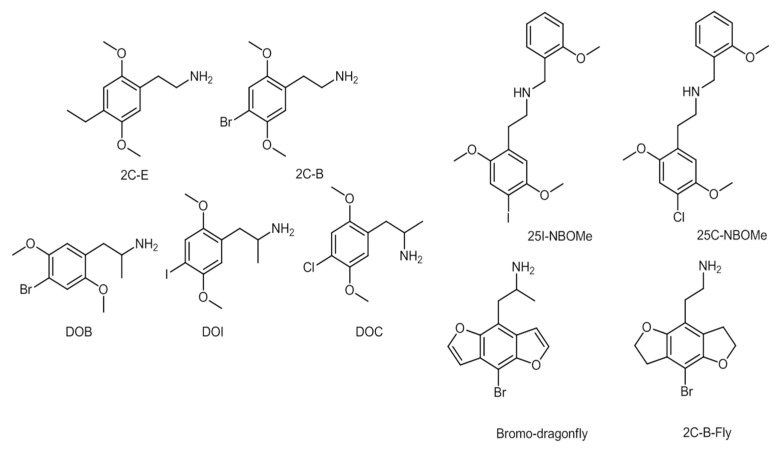
Chemical structures of several phenylethylamines.

2C series substances include compounds with aromatic ring substitutions such as [2-(4-ethyl-2,5-dimethoxyphenyl) ethanamine] (2C-E) or 2C-B. D series substances include amphetamines with aromatic substitutions, such as [1-(4-iodo-2,5-dimethoxyphenyl)propan-2-amine] (DOI) or [2,5-dimethoxy-4-chloroamphetamine] (DOC). NBOMe series contain an *N*-methoxybenzyl group, featuring compounds such as [2-(4-iodo-2,5-dimethoxyphenyl)-*N*-[(2-methoxyphenyl)methyl]ethanamine] (25I-NBOMe) or [2-(4-chloro-2,5-dimethoxyphenyl)-ı-[(2-methoxyphenyl)methyl]ethan-1-amine] (25C-NBOMe). The benzodifurans encompass a range of compounds, such as [1-(4-bromofuro[2,3-*f*][1]benzofuran-8-yl)propan-2-amine] (bromo-dragonfly) and [2-(4-bromo-2,3,6,7-tetrahydrofuro[2,3-*f*][1]benzofuran-8-yl)ethanamine] (2C-B-Fly), while other compounds belonging to the phenylethylamines’ group are, for example, PMMA [11,153]. The chemical structures of a few phenylethylamines are presented in Figure 10 [11,153].

Several substances from the referred series are yet to fall under international control, being regulated only in a few countries [11]. An important and relevant aspect of the consumption of phenylethylamines is shown in the NBOMe series compounds. These substances are often sold as LSD, but have higher toxicity [153].

The consumption method varies with the specific type of phenylethylamines. Ingestion is the most common, but phenylethylamines can also be insufflated, or taken as pills or capsules. Substances such as the 2C series are found in other forms such as powders, liquids, and tablets [154]. Phenylethylamines were the third most reported group of NPS worldwide (437 cases), right after synthetic cathinones and synthetic cannabinoids (684 and 665 cases, respectively) [12].

Phenylethylamines are capable of inducing hallucinations, such as methamphetamine, cocaine, and other drugs, as they mediate activity of several amine receptors [12]. In fact, a study from 1996 showed that DOI, a hallucinogen, acts as a partial agonist of 5-HT_2A_ receptors, involved in mediating the hallucinogenic effects of several drug compounds such as LSD [155]. Compounds from the NBOMe series, such as 25I-NBOMe, have also been recognized as agonists of 5-HT_2A_ receptors [156].

Phenethylamines derivatives can also be used for therapeutic purposes, since they can affect several systems, such as the serotoninergic, dopaminergic, and noradrenergic [157]. Thus, these substances can be administered as appetite suppressants, vasoconstrictors, bronchodilators, or calcium channel blockers [157,158], and can be sold as stimulants, hallucinogens, anti-depressants, anorectics, hormones, neurotransmitters, or bronchodilators [157,159].

Concerning adverse effects, compounds from the D series have been reported as potential vasoconstrictors and induce tachycardia, seizures, hallucinations, kidney failure, and others [11,160]. As for the NBOMe series, adverse effects include cardiovascular problems, seizures, metabolic acidosis, and organ failure. As for the 2C series, various clinical pictures have been reported for different patients, such as serotonin toxicity or sympathomimetic syndrome [161]. The symptoms are similar to other phenethylamines: hallucinations, euphoria, nausea, tachycardia, and respiratory depression, for example [161,162].

Little information is available concerning phenylethylamines’ metabolism in humans. Several factors may influence the pharmacokinetic profile of these substances, namely the user’s tolerance and toxicity or purity of the compounds [154,163].

Theobald and Maurer [164] identified the metabolites of 2C-E in rat urine by gas chromatography coupled to mass spectroscopy (GC–MS). The authors reported that the pathways responsible for the metabolization of the compounds are *O*-demethylation, *N*-acetylation, and hydroxylation, and identified metabolites such as *N*-acetyl-2C-E and trifluoroacetylated 2C-E.

As for the NBOMe series compounds, a few studies in vivo and in vitro have indicated that the main metabolic pathways are *O*-demethylation, hydroxylation, and *N*-demethoxybenzylation for different compounds within the series [165,166,167]. A few examples are described in a study performed by Šuláková et al. [165], which identified the compounds hydroxy-25CN-NBOMe (isomer 2), dehydro-25CN-NBOMe, and *O*-demethyl-25CN-NBOMe in human liver microsomes.

The consumption of phenylethylamines poses a serious threat to public health worldwide due to their toxicity. Serotoninergic syndrome, for instance, is one of the main dangers of phenethylamines consumption [168].

Several case reports concerning hospitalizations or fatalities have been published in the literature. Bromo-dragonfly has been linked to several deaths in Scandinavia [11]. Compounds from the 2C series such as [2-[2,5-dimethoxy-4-(propylsulfanyl)phenyl]ethan-1-amin] (2C-T-7) have been linked to three fatalities in which a drug cocktail was ingested [169]. PMA and PMMA are the most common phenylethylamines to be associated with fatalities [12].

Concerning specific cases, Stellpflug et al. analyzed samples from a non-fatal case of an 18-year-old woman that was admitted to the emergency department with seizures, tachycardia, agitation and hypertension associated with the consumption of 25I-NBOMe [170]. Still concerning this compound, a fatal case of a 19-year-old man was also reported [171]. Lastly, a recent example concerns a fatality of a 17-year-old man caused by the ingestion of 25B-NBOMe [172].

### 2.5. Tryptamines

These substances are structurally similar to serotonin [173]. Tryptamines have high affinity for 5-HT receptors, and the induced hallucinations are mostly mediated by the 5-HT_2A_ receptor [174]. Some of these compounds also release dopamine, serotonin, and norepinephrine [175].

Tryptamines occur naturally in plants, fungi, and animals [173]. They play a very important role in the human body, since serotonin is a naturally-derived hormone involved in regulating the CNS, operating in the regulation of sleep, cognition, memory, temperature, and behavior [176].

Ayahuasca is a hallucinogenic drink made from a plant called *Banisteriospsis caapi*, or an association with *Psychotria viridis*. [22,177]. The leaves of the latter are rich in DMT, a tryptamine that also exists in other plants. This drink is normally used by the indigenous tribes of the Amazon jungle in religious rituals, and are also used to treat depression, anxiety, alcohol, tobacco, and drug addiction [22,23,177].

Other substances of this group can be found in *Psilocibo* spp. fungi, a type of mushrooms widely distributed that has been used by indigenous people for sacred and therapeutic rituals in South America, India, Mexico, and Australia. These mushrooms contain two main compounds: psilocybin and psilocin [174,178]. These two substances can be found in about 190 species of Psilocybe mushrooms [173]. Psilocybin and psilocin have similar properties to LSD, the reason why they became known worldwide as “magic mushrooms” [22,178]. Psilocybin is also known for its therapeutic potential, being effectively used in the treatment of resistant depression, as well as in the treatment of anxiety and depression in cancer patients [178,179]. Moreover, its therapeutic use has been suggested as effective and safe for treating alcohol dependence and for tobacco smoking cessation [180].

Another example of a natural hallucinogenic substance in this category is 5-hydroxy-*N,N*-dimethyltryptaline (5-OH-DMT), a positional isomer of psilocin. This compound and its derivative 5-methoxy-*N*,*N-*methyltryptaline (5-MeO-DMT) are the main psychoactive elements present in the venom of the American desert toad *Bufo alvarius* [174].

Lysergic acid amine or (8β)-9,10-didehydro-6-methyl-ergoline-8-carboxamide (LSA), an analogous to LSD, appears naturally in the seeds of *Argyreia nervosa* and *Ipomoea violacea*. The similarity in the structures of LSA and serotonin has triggered interest for the application of LSA in the therapy of mental disorders and treatment of alcoholism [174].

These natural compounds attracted attention of drug developers, who, by changing their chemical structure, created NPS. Many of these new compounds are not objects of animal or human studies, their acute or long-term effects are not known, and neither are their possible interactions with other substances or toxicological risks; this lack of information poses a public health risk. [174]. These drugs are sold worldwide through the internet, and are used as cheaper substitutes for classic hallucinogens [175].

**Figure 11:**
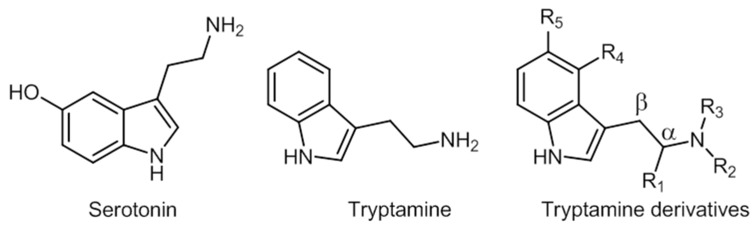
Chemical structures of serotonin, tryptamine, and tryptamine derivatives.

Serotonin and tryptamine are structurally very similar (Figure 11).

Tryptamines derive from the decarboxylation of tryptophan. This produces the indole ring typical of these compounds, which gives them the name of indolealkylamines [173].

Minor additions and modifications to the structure of indolealkylamines provide a virtually infinite supply of new tryptamine structures. Table S3 (Supplementary Material) summarizes some of the most common synthetic and natural compounds of this family.

**Figure 12:**
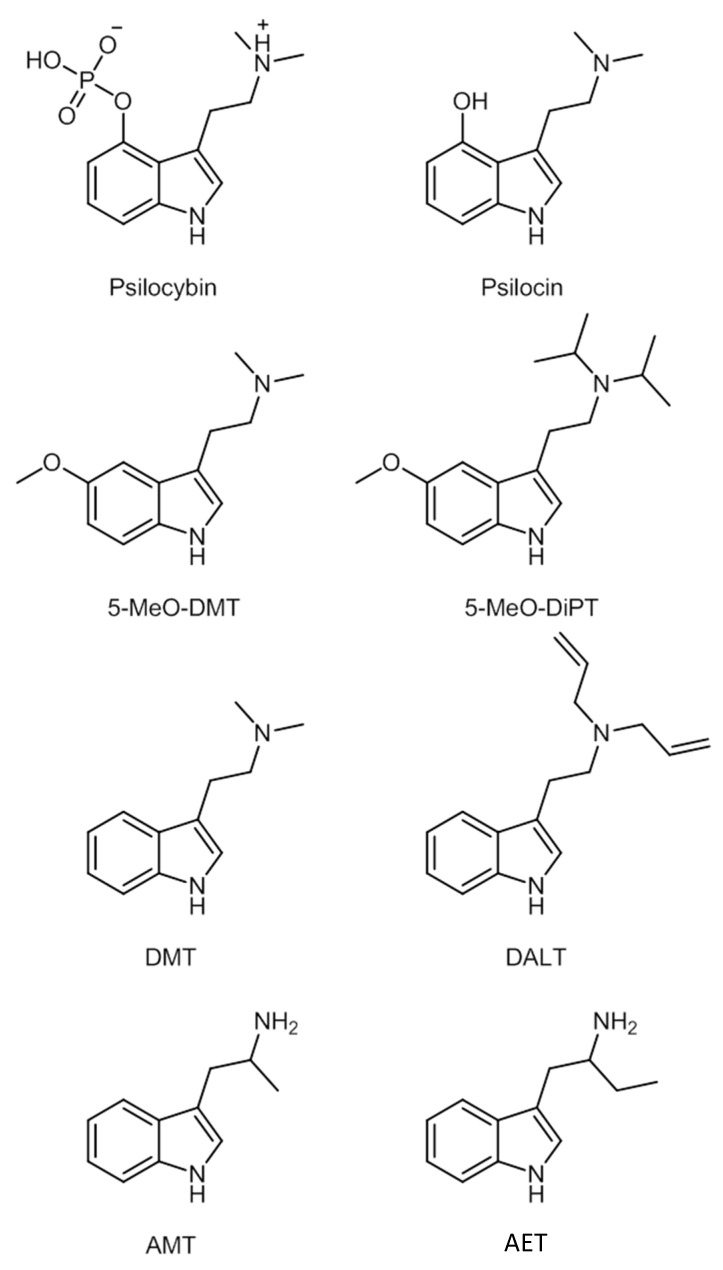
Examples of tryptamines.

The main characteristic of the structure is the indole moiety of the molecule, which is responsible for the hallucinogenic properties. The changes in positions R4 and R5 are those that tend to create most compounds [174]. Some of these compounds’ chemical structures are represented in Figure 12.

There is currently little information on the metabolic pathways or enzymes involved in the metabolization process of tryptamines [173]. Concerning LSD, less than 1% is eliminated unchanged in urine, being widely metabolized. Five metabolites were observed in urine: 2-oxo-LSD, 2-oxo-3-hydroxy-LSD, *N*-demethyl-LSD, and 13- and 14-hydroxy-LSD glucuronide. Psilocybin is dephosphorylated, originating from psilocin, its active metabolite. It subsequently undergoes new metabolization to form 4-OH-IAA (acetic 4-hydroxyindol acid) and psilocin *O*-glucuronide [174].

Other tryptamine derivatives such as 5-MeO-DMT, 5-OH-DMT, and DMT undergo MAO-A mediated metabolization. The metabolic pathway of DMT catalyzed by MAO-A is not the only one. Some metabolites have been found in human urine and blood, such as *N*-methyltryptain (NMT), 2-methyl-1,2,3,4-tetra-hydro-beta-carbolin (2-MTHBC), and 1,2,3,4-tetra-hydro-beta-carbolin (THBC). These are, however, minor metabolites. DMT-*N*-oxide (DMT-NO) is a biotransformation product by *N*-oxidation, *N*-demethylation, and cycling, after oral administration of ayahuasca [174].

When administered orally, substrates for MAO are rapidly metabolized, losing their hallucinogenic activity. This leads users of DMT and 5-MeO-DMT to also consume MAO inhibitors. Thus, ayahuasca also contains β-carbolines to obtain this effect. The metabolic pathway of MAO is reduced, increasing the psychoactive effect of these compounds [174].

5-Methoxy-diisopropyltryptamine (5-MeO-DiPT), a recent derivative of tryptamin, is metabolized by three distinct metabolic pathways. The first is *O*-demethylation; the second by hydroxylation and methylation; and the third by *N*-desalkylation [174].

The easy access to these substances is a public health problem, as they are available on the internet, in nightclubs and raves, which seems to be a growing market. From insignificant to significant quantities, different substances are being sold worldwide, and usually, users do not know what they are buying [173].

Psilocybe mushrooms are usually consumed as infusions or eaten raw. The dose of psilocybin per mushroom varies, and so do the effects [174].

Tryptamines can be taken orally, intramuscularly, or intravenously, but they can also be smoked [176]. These different routes of administration depend on the substance or on the consumer’s preference [174]. For instance, DMT, is not active orally, so it is usually smoked or administered intramuscularly, subcutaneously, or intravenously [176].

The route of administration influences the onset and duration of action. Psilocybin has an onset of action of 20–40 min and a duration of 4–6 h when administered orally, but when administered intravenously, this onset is much faster (1–2 min), and the effects last up to 20 min [173]. Tryptamines are known to have a short duration of action, which encourages repeated consumption. This results in higher consumption habits, increasing the risk of dependence [173].

The popularity of synthetic tryptamines has been increasing due to their similarity to LSD at a reduced cost [174]. According to the 2019 Global Drug Surveyreport, about 40% of drug users consume these substances, and tryptamines are the class with the highest increase in use. The most used substance in this family is LSD (17.5%), followed by psilocybin, or magic mushrooms (14.8%), DMT (4.2%), magic truffles (3.3%), and ayahuasca (1.1%) [173].

The European Drugs Report states that the prevalence of LSD and magic mushrooms among young adults (15–34 years) in the EU has values below 1% for both substances. However, there are exceptions for psilocybin mushrooms. Finland (2%), Estonia (1.6%), and the Netherlands (1.1%) are the countries with the highest prevalence of this drug. Concerning LSD, the country with the highest prevalence of use is Finland (2%), followed by Estonia (1.7%) [115].

As also happens with other NPS, the chemical constituents of the product are rarely fully described, or the information may be incorrect. Thus, there are risks associated with consumption and a possibility of overdose upon repeated administration [173].

The effects of tryptamines include visual hallucinations and mental and perception changes, such as hypersensitivity, perception of physical and temporal space, feeling of unreality and altered personality, as well as distortions, illusions, and auditory, visual, and sensory hallucinations [174].

Other neurological effects may also occur, such as clonus, ataxia, hyperreflexia, agitation, psychosis, delusions, paranoia, excitability, anterograde amnesia, cataplexy, and confusion. They can also induce tremors, seizures, and panic reactions (which can be called “bad trips”), depressive psychotic effects that usually occur in consumers with existing psychopathologies [174].

Hallucinations or changes in perception can appear days, months, or even years after tryptamine consumption (“flashbacks”) [174].

Tryptamine users may exhibit physiological health changes, such as increased heart rate, hypertension, tachypnea, hyperthermia, rhabdomyolysis, renal failure, trismus, euphoria, anxiety, diarrhea, abdominal cramps, sweating, vomiting, palpitations, drowsiness, dysphoria and mydriasis [174].

There is no evidence that the consumption might pose a life-threatening risk due to cardiovascular, renal, or hepatic changes, as these substances do not have much affinity for the receptors present in these systems [174].

However, when the body is exposed to both tryptamines and IMAO, harmful results may occur. By prolonging the effects of tryptamines due to the inhibition of MAO, hyperserotonergic effects may occur, as well as serotonin toxicity. These phenomena occur due to the agonist behavior of tryptamines and IMAO [174].

Although not commonly directly associated with death, risks can trigger situations and induce behaviors that lead consumers to put themselves at risk [174].

Some data reveal that the use of these drugs may be beneficial for the treatment of psychiatric conditions, emotional stress, tobacco dependence, and depression. LSD (34.1%), magic mushrooms (20.4%), ayahuasca (3.9%), DMT (2.4%), and 5-MeO-DMT (0.2%) are the most commonly used tryptamines in these cases [181].

Bilhimer et al. [182] reported a case of a 25-year-old man who was submitted to the emergency room after consuming a tea purchased on the internet, that contained ayahuasca. Laboratory findings indicated a Creatine Kinase concentration of 895 IU/L, a white blood cell count of 20 K/mm^3^, and positive urine immunoassay for amphetamines. A later analysis showed a concentration greater than 2000 ng/mL in the urine sample.

Honyiglo et al. [183] reported a case of an 18-year-old French man who ingested magic mushrooms. The victim assumed unusually aggressive and excited behavior and had an increased urge to jump off the balcony, which he followed up with and died. The autopsy results determined that the cause of death was multiple trauma, secondary to an accidental high fall under the influence of psilocybin mushrooms. The toxicological analysis in *postmortem* samples revealed that consumption around 5 g of dried magic mushrooms had occurred a few hours before death. A total psilocin concentration of 2.230 ng/mL was found in urine samples, 60 and 67 ng/mL in peripheral and cardiac blood respectively, bile (3102 ng/mL), and vitreous humor (57 ng/mL). In addition to psilocin, other substances such as THC (1.34 ng/mL), OH-THC (0.53 ng/mL), and THC-COOH (1.88 ng/mL) were found.

Attema-de Jonge reported the case of two young men, 25 and 32 years old, who showed signs of self-mutilation with knives after consuming psilocybin mushrooms. One of them also ingested cocaine, cannabis, and alcohol, which together with these tryptamines can increase the risk of dangerous behavior [184].

Sklerov et al. [185] reported a case of a 25-year-old man, who was found dead the morning after the consumption of an extract of herbs containing β-carbolines and hallucinogenic tryptamines. Toxicological reports identified DMT, tetrahydroharmine, harmaline, harmine, and 5-MeO-DMT, which was the substance with the highest concentration.

### 2.6. Synthetic Cannabinoids

Synthetic cannabinoids, also known as synthetic cannabinoid receptor agonists (SCRA), were laboratory created, with intention for use as therapeutic pharmaceuticals [186]. Several compounds were synthesized during the 1970s of the last century by the pharmaceutical industry and academic/research laboratories, and their names came from the person or institution responsible; for example, the JWH family was synthesized by John W. Huffman, while HU-210 was synthesized at the Hebrew University in Jerusalem. More than 450 SCRA were synthesized over the course of 20 years [186], but their recreational use only emerged in the 2000s [187].

**Figure 13:**
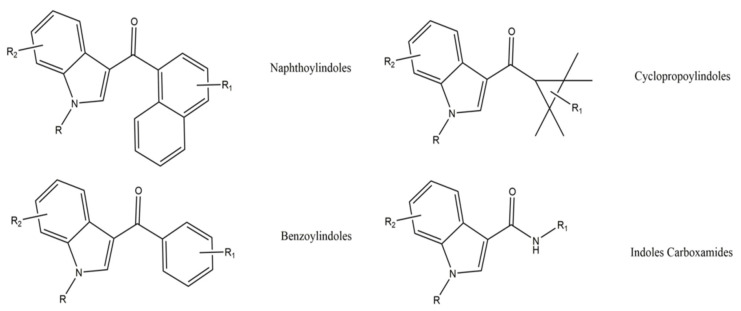
General chemical structures of some aminoalkylindoles subgroups: naphthoylindoles, benzoylindoles, cyclopropoylindoles, and indoles carboxamides.

Nowadays, these substances are mostly classified according to their chemical structures [188]. According to the EMCDDA, seven major groups of SCRA exist, namely naphthoylindoles, naphthylmethylindoles, naphthoylpyrroles, naphthylmethylindenes, phenylacetylindoles, cyclohexylphenols, and classical cannabinoids [189]. SCRA classification is summarized in Supplementary Material Table S4. Structural changes are made to SCRA molecules forming new substances. Therefore, it is natural that alterations made on a single molecule can originate several others. Inside the aminoalkylindole group (Figure 13), it is possible to find other subgroups, such naphthoylindoles, where changes made in the R group can originate from substances of the JWH family, for example JWH-018 and JWH-073, among others. On the other hand, if alterations are made on the central molecule of the benzoylindoles subgroup, it is possible to obtain RSC-4 or AM-694. In the same way, if the R or R1 is modified in the indole carboxamide subgroup, we can obtain APICA or even 5F-APICA substances.

Information concerning the classification of SCRA can be found in the UNODC document about recommended methods to identify these compounds in seized materials [190].

These compounds are sold illegally, usually added to plant materials (crushed leaves), wrapped in aluminium foil—herbal mixtures—but can also be sold in a solid or oily form, if in their pure state [189]. Comparative to cannabis, these substances are essentially smoked, but oral use has been reported, and less commonly, injections. Recently, e-liquids are also available [191,192,193,194]. It is common, in prison surroundings, to soak papers or tissues with SCRA, and then smoke them with tobacco or vape them using electronic cigarettes [195,196]. Nowadays, it is possible to acquire these substances on online markets (darknet), through drug “dealers”, or even by exchanging products with other consumers [196].

An UNODC report from 2021 indicated that 29% of NPS monitored from 2010 to 2020 were SCRA [197]. In the EU alone, 209 new SCRA were identified from 2018 to 2020 [198]. Such numbers pose a major concern in terms of legislation and public health, because most of these compounds are synthesized with impurities, contaminants, and adulterants [188,199]. The same UNODC report showed that 16% of fatalities associated with NPS, were due to the use of SCRA. These substances are mainly used by adolescents and young adults, due to their interest in cannabis [200,201,202].

Chemically, SCRA are structurally different from THC. Still, they are lipophilic and nonpolar [188]. Knowing pharmacokinetics and pharmacodynamics properties of SCRA is key to further understand the obtained results from case reports and others [203]. SCRA produce physiological and psychotropic effects, varying in duration and severity. However, little is known about the pharmacology and toxicology of them and metabolites, with few studies made in humans [188]. SCRA metabolism began to be under focus in the early 2000s [204,205,206]. In the same decade, Sobolevsky et al. were able to first identify JWH-018 metabolites in human urine samples of three individuals who confessed to have smoked at the very maximum 1 g of *“Tropical Synergy”* [207]. Authors were able to detect 13 metabolites and concluded that these metabolites, even the phase I metabolites and phase II metabolites, are excreted through glucuronidation reactions [207]. It is important to trace metabolites, since JWH-018 is not detected in urine [208]. Recently, Toennes et al. [209] also studied the metabolism of JWH-018 in human serum samples. By using a 2 mg and 3 mg dose of this substance, the estimated half time of distribution is 0.40 h and 0.45 h, respectively. In addition, concentrations in JWH-018 reached their maximum blood concentration within minutes after intake. In this work the authors were able to detect JWH-018 pentanoic acid, JWH-018 *N*-(3-hydroxypentyl), JWH-018 *N*-(4-hydroxypentyl), and JWH-018 *N*-(5-hydroxypentyl) metabolites in urine. Moreover, the t_1/2_ of distribution of the studied metabolites ranges from 0.61 to 1.23 h, when consuming a 2 mg dose of JWH-018 and 0.58 to 1.10 h when 3 mg of the same substance was inhaled [209]. In fact, glucuronidation is the preferred route for excretion of these substances, originating their metabolites [210,211,212]. Furthermore, human uridine diphosphate-glucuronosyltransferase (UGT) is responsible for this mechanism [211]. Kong et al. [213] and Abbate et al. [214] summarize the metabolism of JWH-018 and Chimalakonda et al. [215] describe the metabolism of JWH-018, as well as AM-2201 with its main metabolites. In a study about the metabolism of 5F-AB-P7AICA, Giorgetti et al. [216] proposed a metabolic pathway. After self and controlled administration, it was possible to detect a peak of the compound in urine samples, suggesting that this compound can be monitored without considering its metabolites. Moreover, 10 metabolites were confirmed in urine. The parent compound originated from hydroxylated, dehydrogenated compounds, as well as the metabolites originated from hydrolytic defluorination and amide hydrolysis [216].

Cannabinoids and SCRA exert their effects on the endocannabinoid system, mainly in the cannabinoid receptors 1 and 2 (CB_1_ and CB_2_), yet with higher binding affinities [217].

Data are limited about adverse effects and intoxications associated with the consumption of SCRA, because of their constant structural changes, which culminates in different potencies, efficacies, and duration of action. Nonetheless, studies were able to demonstrate effects of such compounds, for instance, seizures, tachyarrhythmia, anxiety, hallucinations and confusion, mydriasis, drowsiness, hypo and hypertension, and vomiting. [218,219]. Headaches, slowed speech, sweating, aggressiveness, lethargy and slowed speech have also been reported for other SCRA [220]. Cardiovascular effects as arrhythmias, cardiac arrest, cardiomyopathy, coronary thrombosis, acute myocardial infarction, and vasculitis have been reported [221,222,223,224]. A 34-year-old male died after consuming 5-Fluoro-ADB and was submitted to autopsy [225]. The target compound was found in various matrices, including adipose tissue and heart muscle, but not in urine or blood fluids. The highest concentration was reported in adipose tissue, 7.95 ng/mL, followed by stomach and brain, with 3.18 ng/mL and 1.90 ng/mL, respectively. Less than 0.5 ng/mL was reported in lung, liver, and skeletal muscle. After inhalation, and loss of consciousness, death was due to cardiopulmonary arrest. A year later, the same authors were able to perform a trial identification of 5-fluro-ADB-PINACA and MAB-CHMINACA, except for urine, in which MAB-CHMINACA was not detected. Concentrations found ranged from 6.05 ng/mL to 156 ng/mL, the highest concentration was detected in liver tissue, followed by kidney and pancreas [226]. Minakata et al. [227] determined 6 SCRA (AB-PINACA, AB-FUBINACA, AB-CHMINACA, MAB-CHMINACA, and 5F-AMB NS 5F-ADB) in the urine of three different cadavers. AB-PINACA and AB-FUBINACA were found in victim 1, with concentrations of 23 pg/mL and 10 pg/mL, respectively. Moreover, 239 pg/mL of AB-CHMINACA was detected in victim 2 and 229 pg/mL MAB-CHMINACA in victim 3. 5F-ADB was found both in victim 2 and 3, with concentrations of 19 pg/mL, each [227]. Three fatal cases involving 5F-ADB, 5F-PB-22, and AB-CHMINACA were reported by Angerer et al. [228]. In this case, it is widely believed that death was due to consumption of these substances, as well as concomitant use of ethanol in two of the cases. In the other, toxic effects of antidepressants and 5F-ADB are thought to be the cause of death [228]. Another study hypothesized that SCRA consumption could be related to diabetic ketoacidosis and ultimately death [229]. Blood samples were collected and AB-CHMINACA, AB-FUBINACA, AM-2201, 5F-AMB, 5F-APINACA, EAM-2201, JWH-018, JWH-122, MAM-2201, STS135, and THJ 2201 were detected by liquid chromatography tandem mass spectrometry (LC–MS/MS). The 25-year-old victim had a drug consumption history. Apart from the mentioned SCRA and medicinal drugs for diabetes (insulin), no other substances were detected. Allegedly, the cause of death was due to SCRA consumption and the lack of consumption of insulin, since high levels of glycosylated hemoglobin and acetone were detected in blood [229].

### 2.7. Phencyclidine Analogs

**Figure 14:**
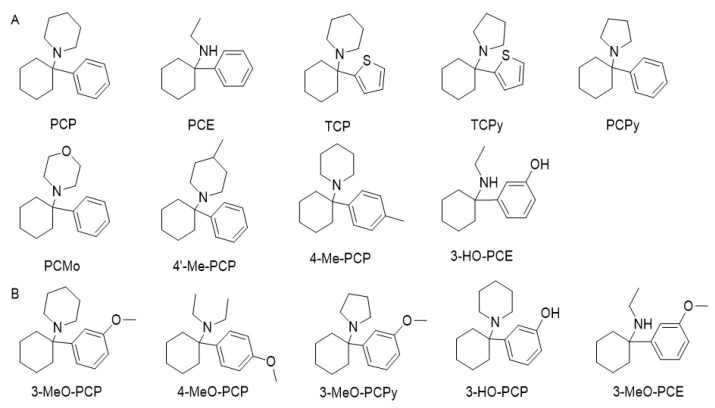
(**A**) Phencyclidine (PCP) and examples of first-generation analogues that appeared on the streets between the 1960s and 1990s. (**B**) Phencyclidine analogues that appeared as a new psychoactive substance in recent years.

Phencyclidine-like substances belong to the arylcyclohexamines family, a group of chemicals considered to be dissociative hallucinogens, which have structures similar to those of PCP and ketamine (Figure 14).

PCP was first synthesized in 1956 as an intravenous anaesthetic for humans, being approved by the FDA in 1957 and sold until the late 1960s. However, it was withdrawn from the market due to adverse psychological effects that were often disturbing and sometimes severe and prolonged [230,231,232,233,234,235]. It was reintroduced as an animal tranquillizer in the late 1960s [232,234,236]. The first report of recreational use of the drug was in the late 1960s in San Francisco under the name of “peace pill” [236]. Even though PCP was the first synthesized arylcyclohexylamine, other compounds had been reported before. For example, 1-(1-phenylcyclohexylamine)amine (PCA) was first stated in 1907, in 1953 *N-*ethyl-1-phenylcyclohexylamine (PCE) was investigated, and 1-(1-Phenylcyclohexyl) morpholine (PCMo) in 1954 [231].

Approximately 14 analogues were reported between 1960 and 1990. Since then, many more have been published (Supplementary Material Table S4). Still, the use of three compounds was prominent, considered to be in the first generation of phenylcyclohexyl analogues: PCE, TCP, and 1-(1-Phenylcyclohexyl) pyrrolidine (PCPy) [230,231,236,237].

The use of PCP has declined since the 1980s. Although it remains popular and its consumption is increasing, especially for nightclub attendees, it essentially is confined to the United States and Canada, with occasional reports in the European Union [230,231,233]. According to the Drug Abuse Warning Network, in 2011, there were 75,538 emergency department visits, most of which were due to combinations with other drugs, such as marijuana, cocaine, painkillers, and anxiolytics. Two deaths from PCP were reported in 2012, according to the American Association of Poison Control Centers [232,238].

To date, there is no international protocol for controlling these drugs. Nonetheless, there are specific European countries (e.g., France or Germany) with laws that banned PCP by the narcotics act [239,240]. Since phencyclidine-type substances are most common in the United States, PCE, PHP, PCPY, and TCP are controlled in Schedule I of the 1971 Convention [18], while PCP, PCC, and PCA are controlled in Schedule II of the 1978 Convention [231].

A critical review on ketamine and other phencyclidine analogues was published by Vargas et al. [235].

Ketamine was created in 1962, and subsequently commercialized in 1969. There is no evidence of long-term neurotoxicity or prolonged unfavorable psychological effects when used in a controlled clinical setting. This analogue is popular worldwide and has several therapeutic applications, including general anaesthesia, analgesia, depression, psychiatric treatment, and veterinary use [231,234,236,237,241]. In 2019, the FDA approved a new nasal spray medication for the treatment of depression, containing esketamine, an *S*-enantiomer of ketamine [242].

The first report of ketamine use for its psychedelic effects was in 1971. Illicit ketamine is commonly named “Special K”,” Ket”, “vitamin K”, and is often found as a white crystalline powder or pharmaceutically packaged injectable solution and is usually inflated, injected, or less commonly, consumed orally [231]. It is often adulterated by the addition of caffeine, MDMA, heroin, or cocaine [236]. Supplementary Material Table S6 shows a study with 126 patients without any fatal cases, but with different levels of toxicity [236]. Ketamine is highly lipid-soluble; therefore, distribution to the CNS is enabled. It has a half-life of 3 h, binding affinity of about 10–30% to proteins and its oral bioavailability is 17% [236]. The onset of action of ketamine and methoxetamine (a new derivative) is rapid following intravenous and intramuscular administration, with effects apparent after 1 min and 5 min, respectively. After nasal insufflation, the onset of action is up to 30 min, being up to 30–90 min. for methoxetamine [236].

Ketamine is metabolized primarily by the CYP2B6 isoenzyme, with contributions from CYP3A4 and CYP2C9. Ketamine’s main metabolic pathway is *N-*demethylation to norketamine, its primary metabolite, which has one-third the potency of ketamine. Norketamine undergoes hydroxylation on the hexanone ring, and subsequent glucuronidation to more water-soluble metabolites that are excreted in the urine and also in the bile [236].

The increasing reports of urotoxicity associated with the chronic use of ketamine in high doses have led to the emergence of methoxetamine [231,236,243].

**Figure 15:**
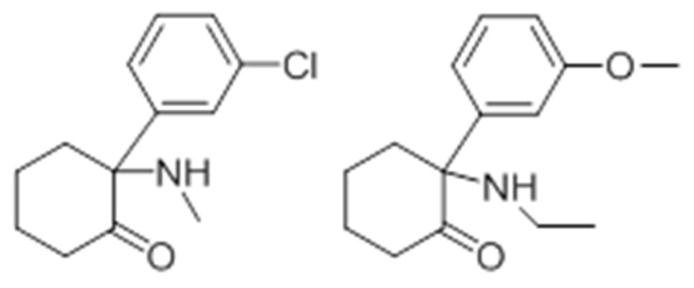
Chemical structure of ketamine and methoxetamine.

Methoxetamine (Figure 15) is a derivative of ketamine and has similar effects; therefore, the potential for abuse is also high. It has been suggested that methoxetamine may share the same neuropharmacological properties as NMDA antagonist and dopamine reuptake inhibitor [243].

Methoxetamine first appeared in 2010 and was widely marketed by “major stores” on the internet, which led to several reports of hospitalization [231,236,243]. Several routes of administration are possible, including intramuscular, oral, inhalation, rectal, or intravenous. So far, the metabolism of methoxetamine is not completely known, but it appears that CYP2B6 and CYP3A4 are involved. The primary metabolite, normethoxetamine, results from *N-*deethylation and undergoes hydroxylation and glucuronidation to form the more water-soluble hydroxynormethoxetamine glucuronide. These metabolites are consistent with those found in urine samples from analytically confirmed methoxetamine cases [236].

Both ketamine and methoxetamine have similar effects, considered positive effects such as euphoria and having a sense of calm and serenity, neutral effects such as distortion or loss of sensory perception, and harmful effects such as severe dissociation, depersonalization, loss of consciousness, nausea, and vomiting [243,244]. According to Botanas et al. [245], MXE and enantiomers have antidepressant effects via glutamatergic and serotonergic mechanisms, as also happens with ketamine,.

The synthesis of 4-MeO-PCP dates back to 1965 as part of a systematic search for PCP analogues with potential clinical use [230,236,240]. The first signs of underground 4-MeO-PCP experimentation began to surface in 1999, but it was not until about 2008 that it arrived in online shops as one of the first dissociative drugs [230]. 4-MeO-PCP is available as powder or tablet, is active via the oral and parenteral routes, and is reported to induce dissociative effects, but with substantially reduced potency relative to PCP and 3-MeO-PCP [230,233,240]. They also present appreciable affinities for the serotonin transporter and high affinities for sigma receptors [240]. The dosage of 4-MeO-PCP is much higher compared to others, as the usual oral dose can be up to 250 mg. This difference in drug activity between isomers can be responsible for acute and severe intoxication in case of confusion by addicts [240]. 3-MeO-PCP became available online as a research chemical in 2011 [233,236,240]. It is known that 3-MeO-PCP is a more potent NMDA receptor antagonist than PCP, presenting almost three times the affinity [231,233,236]. They also present appreciable affinities for the serotonin transporter and high affinities for sigma receptors [240]. 3-MeO-PCP is available as powder or tablets and is commonly administered orally but can also be injected, snorted, or smoked. Sublingual, intramuscular, and rectal administration have also been reported [231,233,240,246]. Elimination has been estimated to be approximately 11 h [230]. Abuse doses are usually between 5 to 20 mg. Duration of effects has been reported to be of approximately 4.5 h, with onset within 30 min after oral ingestion and a peak at approximately 2 h [233]. It produces many adverse effects, including psychosis, confusion, violent behavior, hypertension, tachycardia, suicidal impulses, and coma to death [240].

### 2.8. Synthetic Opioids

Opioids cause anesthetic and depressant effects; however, their recreational use has been of public health concern [247]. According to the UNODC report from 2013, up until this date, the only opioid in the spotlight among NPS was *O*-desmethyltramadol (the main active metabolite of tramadol) that was consumed together with kratom [12,248]. Novel synthetic opioids, or simply synthetic opioids, include fentanyl and analogues, but compounds such as tramadol, methadone, pethidine, ketobemidone, levorphanol, dextromoramide, dipipanone, and others are also included in this category. However, the majority of these are already under regulation policies, which is not the case of most abused substances [247]. Amongst recent fentanyl analogues, the WHO highlights acetylfentanyl, butyrylfentanyl (or butyrylfentanyl), acryloylfentanyl (or acrylfentanyl), carfentanil, furanylfentanyl, and ocfentanil [249]. Most common non-medical abused substances include fentanyl analogues, and benzamide opioids, such as AH-7921 and U-47700, and others such as MT-45 and W-18, which are piperazine opioids [250]. Sometimes, these substances can be found in counterfeit opioid pills, or other pills that imitate benzodiazepines, and heroin [251].

Fentanyl was produced in the 1950s [251,252], aiming at finding molecules with good therapeutic properties, but with less undesired side effects [253]. Firstly, it was established to be medically used, as it is still today, for chronic pain treatment and intraoperative analgesia [10,253]. U-47700 and AH-7921 are isomers and were synthetized in the 1970s with the purpose to replace classic opioids, but their production was abandoned due to addictive effects [251]. Although it was first produced in the 1970s, MT-45, or IC-6, first appeared on an EMCDDA report only in 2013 [254,255,256]. Novel synthetic opioids are responsible for the worldwide opioid crisis, with special incidence in the United States [257,258,259]. In fact, from 2009 to 2019, a total of 77 NPS with opioid effects were reported by the UNODC [259]. This phenomenon poses a challenge to public health authorities, as well as legislative authorities to avoid the dissemination, dependence, overdoses, and inappropriate use of these substances.

**Figure 16:**
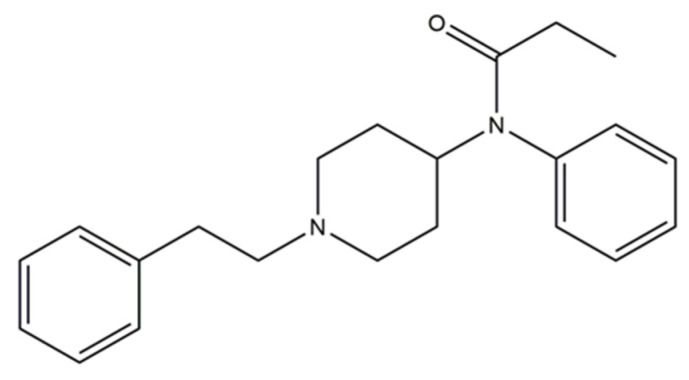
Fentanyl (*N-*(1-phenethylpiperidin-4-yl)-*N-*phenylpropionamide)**.**

Synthetic opioids are basically categorized into fentanyl and analogues, and non-fentanyl structured compounds [260].The former may be further divided into pharmaceutical fentanyls, which include fentanyl, for example, alfentanil and sufentanyl; and non-pharmaceutical fentanyls, which include fentanyl analogues and designer fentanyls, for instance ocfentanyl and bythyrfentanyl [261]. Fentanyl, an analgesic analogue of phenopiperidine, is presented on Figure 16.

**Figure 17:**
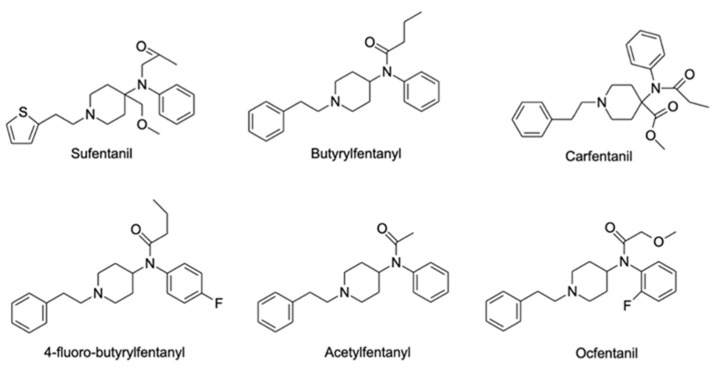
Chemical structures of a few fentanyl analogues: sufentanil, butyrylfentanyl, carfentanyl, 4-fluoro-butyrylfentanyl, acetylfentanyl, and ocfentanil.

Fentanyl analogues (Figure 17) followed fentanyl synthesis and were implemented in veterinary medicine, namely sufentanil, alfentanil, remifentanil, and carfentanil. Other analogues include acetylfentanyl, acryloylfentanyl, α-methylfentanyl, 3-methylfentanyl, furanylfentanyl, cyclopentylfentanyl, and ocfentanil [262]. However, carfentanil is the most used recreationally [251].

**Figure 18:**
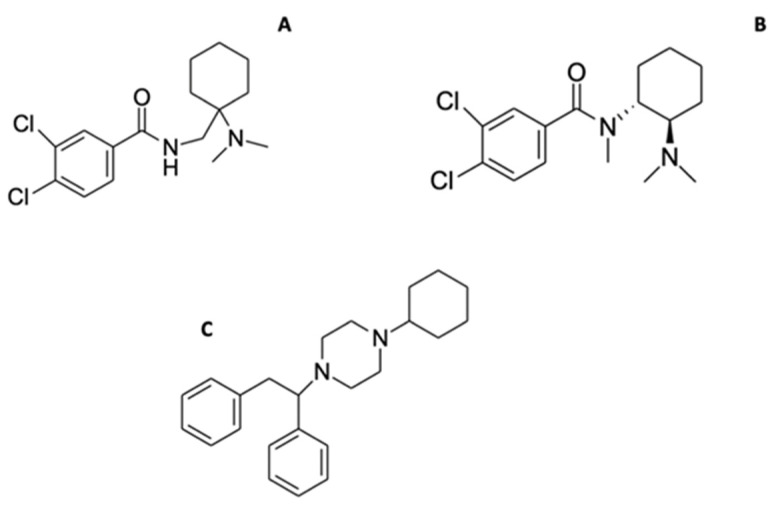
Chemical structures of isomers AH-7921 (**A**), U-47700 (**B**) and MT-45 (**C**).

Substances such as AH-7921, U-47700, and analogues, MT-45, W-18, W-15, and IC-26 (analogue of methadone), are part of the non-pharmaceutical fentanyls family [263,264] (Figure 18). The selling of these new synthetic opioids occurs mostly online [251].

It is not easy to estimate the prevalence of novel synthetic opioids, since the drug market is constantly changing [264]. Oxycodone is a semi-synthetic opioid, used medically, but its recreational use and abuse has been reported [265]. Commonly found in blotters, herbal smoking mixtures, fake medicine tablets or pills, these substances can be swallowed, inhaled (the vapors of heating the tablet), sniffed or even dissolved in water, and injected [266,267,268,269]. Furthermore, fentanyl is also available in the form of patches. Nonetheless, the misuse of such patches can lead to toxicity; rectal insertion is an example [270]. An increase in use of nasal sprays and e-liquids used in electronic cigarettes is also a concern to consider about these substances [271].

From 2009–2020, over 65 new opioids were detected in Europe, and between 2012 and 2020, the vast majority were fentanyl derivatives. Moreover, in 2020, a report from EMCDDA included isotonitazene, a non-fentanyl synthetic opioid that can cause acute respiratory depression, leading to death [115]. This substance was placed under international control in 2021 [197]. In fact, synthetic opioid abuse continues to increase while other substances decline [197]. In 2020/2021, a total of 397 port-mortem cases were reported by the UNODC, and 14% fatalities were due to synthetic opioids. Moreover, 63% of reported cases involving these substances were due to the consumption of acetylfentanyl and 11% to carfentanil. Furthermore, nitazene opioids have recently been reported by the UNODC EWS [197]. Undoubtedly, fentanyl precursors are emerging in the European market, with more than 33 kg of *N-*phenethyl4-piperidone (NPP) seized in Estonia. Other precursors, namely 4-piperidone monohydrate and 1-anilinopiperidine, were also seized in European countries. In 2018, around 70% of the reported opioids were fentanyl derivatives [272]. The consumption of these compounds often leads to death, with few clinical admission scenarios [273].

It is documented that these novel synthetic opioids have a higher potency than morphine [261]. Moreover, opioids are known to wield their effects on opioid receptors, which mediate the actions of exogenous opioids. Similar to SCRA, the mechanism of action on these receptors is through G proteins, thus, mediating adenylyl cyclase by activation or inhibition [274]. Fentanyl and analogues are full agonists of µ-opioid receptors, estimating that fentanyl alone can have a potency of almost 50 to 100 times of that of morphine, while carfentanil has a potency 10,000 times higher than morphine and 100 times higher than fentanyl [251,261]. After intravenous administration of fentanyl, respiratory depression can be reached within 2 to 5 min [275]. The elimination half time of this compound is 219 min. After about 7 to 13 min, maximal serum concentrations are reached, when this compound is administered intranasally, with effects lasting about 1 to 2 h [276,277]. Biotransformation in the liver, by cytochrome P450 isozymes (CYP3A4), originates from norfentanyl [278,279]. These isozymes are also involved in the metabolization of carfentanil (CYP3A5, CYP3A4, CYP2C8, and CYP2C9), forming different metabolites [280].

In 2017, Watanabe et al. [281] predicted the structures of metabolites of acetylfentanyl, acrylfentanyl, furanylfentanyl, and 4-Fluoro-Isobutyrylfentanyl. The authors analyzed human urine samples to perform an in vivo analysis, and incubated the drugs with human hepatocytes for the in vitro analysis. The combination of these analyses allowed for the determination of 32 acetylfentanyl metabolites, a total of 14 acrylfentanyl metabolites and the same number of furanylfentanyl, and 17 4-fluoro-isobutyrylfentanyl metabolites. Metabolites of phase II were mostly observed for acetylfentanyl [281]. Recently, it was possible to identify in vitro ocfentanyl metabolites [282]. A total of four metabolites were produced and results suggested that *O-*desmethylocfentanyl was the most abundant metabolite. A glucuronidated metabolite was also produced [282].

AH-7921 can act as agonist of two opioid receptors (µ and κ) and possesses a high addictive potential [262,283]. There are not many studies about this substance; however, Swedish authorities have reported intoxications and deaths associated with the concomitant consumption of this substance and other psychoactive compounds [283]. In a review article about novel synthetic opioids, Prekupec et al. [284] summarized that AH-7921 opioid can be consumed orally from low to high doses. Its effects last from 6 to 8 h, and after this period, effects can be observed up until 6 h [284]. Furthermore, high doses are considered from 25 mg or higher.

U-47700, the analogue of AH-7921, is considered to have a higher affinity to the µ-opioid receptor when compared to the other opioid receptors [285]. Furthermore, the normal dose of this compound is around 7.5 to 15 mg, and it is approximately 7/8 times more potent than morphine [261,284,286]. MT-45 is an agonist to δ and κ-opioid receptors [256]. When consumed intravenously, it can be 11 times more lethal than morphine [256]. A recent study aimed to obtain a pharmacological characterization of MT-45 in mice [287]. Once again, studies in humans are lacking and they are fundamental to assess the risks of such compounds.

Effects can be observed in the CNS, but cardiovascular and pulmonary effects have also been reported [288].

Sinicina et al. [289] have reviewed *postmortem* cases related to fentanyl. The authors reported that from 2005 to 2014, 242 overdoses in Bavaria, Germany, were related to fentanyl, and, in most cases, this substance was obtained via transdermal patches. The mean concentration in femoral blood was 16.9 µg/L [289]. On a Swedish project called STRIDA, it was possible to observe intoxications due to intake of different novel synthetic opioids (acrylfentanyl—mainly through nasal sprays, 4-fluoroisobutyrfentanyl and tetrahydrofuranfentanyl) [290]. The reported symptoms were anxiety, dizziness, nausea, vomiting, agitation, apnea, asystole, decreased consciousness, respiratory depression, and miosis. A death case due to brain edema is reported, involving the consumption of 4-fluoroisobutyrfentanyl. From the same project it was possible to evaluate intoxications related to butyrfentanyl, 4-fluorobutyrfentanyl, and fentanyl [291]. Recently, Wilde et al. [292], reported a severe intoxication case of cyclopropylfentanyl consumption; the symptoms reported after a 10 min intake were nausea, abundant sweating, and dyspnea, which culminated in miosis, coma, and respiratory insufficiency [292]. Cyclopropylfentanyl was also found in another *postmortem* case. In this case, authors identified that the concentration of this compound increases in femoral blood for 18 h after the collection of the first sample, which suggests *postmortem* redistribution. Intravenous administration of acetyl fentanyl proved to be fatal on a case reported in 2015 by Cunningham et al. [293]. The individual had a history of substance abuse; steroids were detected in urine, and acetyl fentanyl was detected at 235 ng/mL and 234 ng/mL in blood and urine, respectively. Moreover, pulmonary edema and cerebral edema were revealed in the autopsy. Pulmonary edema and lung injury were also observed on a death case associated with the consumption of ocfentanil [294]. Guerrieri et al. conducted research on 40 fatal intoxications in which acrylfentanyl was a contributor or the main contributor to deaths. However, non-fatal cases are also mentioned in the literature [295]. Hikin et al. reported numerous fatalities in the north of England, related to carfentanil, butytyl fentanyl, fluorobutyrylfentanyl, furanylfentanyl, alfentanil and fentanyl, being carfentanil, and fentanyl detected in seven cases. Carfentanil concentrations in blood ranged from 90 to 4004 pg/mL [296]. This same compound was also a target of a study in the United Kingdom and seven deadly cases were reported. The concentrations found in femoral *postmortem* blood ranged from 0.22 to 3.3 ng/mL [297].

Over 6 months, Kronstrand et al. [298] evaluated death cases related to the consumption of AH-7921. Research led to determination of a range of blood concentrations (0.03 to 0.99 µg/g). Although sometimes other compounds were also detected; this suggests that tolerance to this drug can vary, and deaths can occur with low and high doses. Moreover, possible metabolites have also been found [298]. The first reported fatality case with U-47700 was described in 2016 by Elliot et al. [299]. A concentration of 1.46 mg/mL was found in femoral blood, and the metabolites *N-*desmethyl-U-47700 and *N*,*N-*didesmethyl U-47700, and chemical structures were suggested [299].

According to the EMCDDA, MT-45, can cause a wide range of effects, such as, somnolence, unconsciousness, high body temperature and vomiting, as well as tachycardia, decreased respiratory rate, hypoxia, hypo- and hypertension, seizures, low oxygen saturation, and miosis [256]. Other symptoms such as hair depigmentation, hair loss, folliculitis, dermatitis, and dry eyes have also been reported [300].

Overdose due to consumption of MT-45 was considered to be the cause of death in a case reported by Fels et al. [301], and PB-22 and 5F-APINACA were also detected (although in very low amounts). The compound was determined in different matrices, and the concentrations varied between 24 and 1300 µg/mL, with the highest concentrations present in heart blood samples. These concentrations were much higher than those reported in the literature. In a study conducted by Papsun et al. [302], it is reported that MT-45 was involved in one death, together with etizolam, with concentrations of 520 and 35 ng/mL, respectively. The death was attributed solely to the opioid since the concentration of etizolam was therapeutic.

Tabarra et al. [28] recently published an interesting review about these compounds along with their toxicological aspects.

### 2.9. Plant-Based NPS

Plant-based NPS predominantly comprise alkaloids, which can induce a variety of effects, most commonly hallucinogenic, yet some can also induce relaxation [22,114,303]. Most of these NPS are often used for medicinal purposes or in rituals [22,23,304]. Only three plant-based NPS are under monitoring by the UNODC, namely *Catha edulis*, *Salvia divinorum*, and *Mitragyna speciosa* [304,305].

*Catha edulis* (khat) is an indigenous plant from some Middle Eastern and West African countries, and among its contents approximately 40 alkaloids are present, mostly cathedulins (polyhydroxylated sesquiterpenes) and phenylalkylamines [22,23,303,304,306]. Three phenylalkylamines with high stimulant effect were detected: cathinone [S-(-)-cathinone], norephedrine [1R,2S-(-)-norephedrine] and cathine [1S,2S-(+)-norpseudoephedrine]. Khat is often consumed by chewing its fresh leaves, ingestion or smoking being relatively uncommon [303,306]. This is because cathinone is unstable during harvesting and drying processes.

Between 2009 and 2012, this plant was among the most widely consumed NPS in EU member states, being the second most seized plant-based NPS worldwide in 2016. This plant was reported to be frequently misused by synthetic cathinone consumers given its lower risk [22,23,303]. Although the main psychoactive compounds (cathinone and cathine) have been legislated and controlled since the 1971 UN Convention, several European countries, the United States, and Canada have started to introduce restrictions and/or legislation to control the entry of khat in their territories [23,303].

The consumption of *C. edulis* has been associated with psychosis and aggressive behavior, as well as other symptoms, namely irregular blood pressure, tachycardia, urine retention, constipation, and insomnia, although many of these effects are still only described in animal studies [22,23,306].

Several medical conditions such as sleep disturbance, hypotension, and depression can be associated with withdrawal symptoms or a reduction in khat consumption, yet to date, it has not been possible to verify that consumption has been directly responsible for the death of any user [23,303].

*Salvia divinorum* is another of the UNODC-controlled plant-based NPS, and originates from northeastern Mexico, and has been used for centuries for its hallucinogenic properties [22,303,307,308]. This plant is consumed by chewing the leaves, or alternatively consumed as tea or even smoked, being easily purchased in smart shops [22,23,303,304,307,308,309,310].

In the United States, more than 1.8 million people have consumed this substance at least once in their lifetime, with reports from members of the United Nations in 2012 citing the plant as the third most consumed substance of its kind [303,308]. Several European countries, the United States, and Canada have initiated legislation prohibiting consumption or controlling the entry into their territories [22,23,304,308].

The psychoactive effects of *S. divinorum* are promoted by the presence of salvinorin A, a dissociative hallucinogen with selective agonist effect for kappa opioid receptors (KOR), and whose mechanism of action is yet to be properly clarified [22,23,303,307,308]. The effects promoted by salvinorin A are closely related to the route of administration. Psychoactive effects are observed within in a few seconds or minutes after inhalation or consumed through the buccal mucosa, even at low concentrations (200 µg), which can last up to an hour [22,23,303,307,308]. The half-life time of this compound is around 57 min, being longer in women [308].

Short-term effects similar to the consumption of marijuana, LSD, or ketamine are common, including extracorporeal experiences, exacerbated relaxation, colorful visions, and loss of consciousness, yet to date, no deaths have been linked to consumption [22,23,303,307,308]. Salvinorin A has been shown to possess some effects with therapeutic potential, for use in the treatment of drug addiction, pain, neurological and gastrointestinal diseases, and as anti-inflammatory agent [22,309,310].

There has been a growing concern over the recreational consumption of *Mitragyna speciosa* (kratom), a plant native to Southeast Asia [22,23,142,303,308,311,312,313]. It is commonly used for pain relief, to treat withdrawal symptoms in opiate users, hypertension, diarrhea, and coughing [22,23,24,142,303,308,311,312,313]. This plant is usually obtained fresh or dried, frequently consumed by chewing, drinking teas, or smoking.

Several polyphenols, flavonoids, and glycosides have been identified among its constituents, as well as more than 40 alkaloids [22,23,303]. The main alkaloid with psychoactive activity in kratom is mitragynine, followed by 7-hydroxymitragyne, mitraphylline, corynantheidine, speciogynine, paynantheine, and speciociliatine, which are also believed to be associated with the observed effects [22,23,142,303,308,311,312,313].

This NPS is not yet listed in the schedule of the United Nations Convention on drugs. However, *Mitragyna speciosa* is controlled or classified as a narcotic by various entities in some countries in Southeast Asia, Oceania, the United States, and several European countries [22,23,303,311,313].

There is considerable clinical interest in the plant’s analgesic effect, since the affinity of mitragynine and 7-hydroxymitragynine for opioid receptors is well established [22,23,24,303,308,311,313]. This plant has been used for several years in some regions of Asia to reduce fatigue and increase productivity at work, and also to treat coughing, pain, fever, diarrhea, hypertension, and diabetes. Kratom leaves have also been used as substitutes for opium, as well as in morphine withdrawal treatments [22,24]. Mitragynine also interacts with δ and κ receptors, and a partial interaction of 7-hydroxymitragynine with κ receptors has also been reported [22,23,303,308,311,313]. The stimulant effects at low doses are thought to be associated with the same type of mechanism observed in low-dosed morphine [308]. The set of effects begin approximately 5 to 10 min after consumption and may last for up to an hour [303].

Several toxic effects are associated with recreational uses of Kratom, namely irritability, anxiety, and aggressiveness. When consumed for long periods, tremors, anorexia, psychosis, hyperpigmentation of the skin, and weight loss can be experienced [303]. A number of fatalities have been associated with its consumption, with joint exposure to other substances being the most likely cause of death [23,303].

## 3. Conclusions

The circumstances concerning drugs of abuse, and specifically NPS, are constantly changing. New substances capable of producing the effects desired by consumers appear daily, escaping legal control. Due to the relatively low cost and high potency of some of these substances, their use by marginalized groups, for example, people who are homeless or those with long-term and highly problematic drug use, is increasing. Moreover, since new psychoactive substances are more difficult to detect and identify in routine urine tests, they can be used by people who regularly undergo drug testing programs and want to avoid detection of their drug use. Examples of this are phencyclidine derivatives, opiates, and synthetic cannabinoids. Considering that each country has its own legislation on these new substances, different mechanisms exist concerning improving public health or social policies. There is no doubt that EWS plays a central role in supporting readiness and coordinated responses to the NPS problem, both at the national and international level. Awareness campaigns aimed at the younger population highlighting the deleterious effects of these substances (e.g., schools, social media, and media) may also have an important effect. The imprisoned population could also be targeted with such actions. Carrying out studies that identify the mechanism for action of these substances, and their effects, and consequences in the short and long term is undoubtedly a valuable tool in the toxicological field. In addition, laboratorial developments allowing the rapid identification of these substances, mainly at the emergency room level, would help. Furthermore, multidisciplinary research in the areas of epidemiology and prevention, and public health allow the development of adequate measures to monitor consumption and implement public health education policies. All these factors will certainly contribute to the implementation of social and public health measures to efficiently face the scourge of NPS consumption worldwide.

## Data Availability

Data sharing is not applicable to this article.

## Supplementary Materials

The following supporting information can be downloaded at: https://www.mdpi.com/article/10.3390/ijerph19084869/s1, Table S1: Classification of piperazines; Table S2: Intoxications with piperazines; Table S3: Examples of natural and synthetic tryptamines; Table S4: Classes of SCRA and their chemical characterization; Table S5: Phencyclidine-type substances; Table S6: Case report intoxications with phencyclidine-type substances. References [314,315,316,317,318,319,320,321] are cited in the supplementary materials.
