# Supplementary material for: An Update on the Implications of New Psychoactive Substances in Public Health
Source: Int J Environ Res Public Health. 2022 Apr 17;19(8):4869. doi: 10.3390/ijerph19084869 (PMC9028227; doi:10.3390/ijerph19084869)
Supplement: Supplementary file 1 [file ijerph-19-04869-s001.zip › ijerph-1609368-supplementary.pdf]

Supplementary Material:

Table S1. Classification of piperazines

Family	Compound
Benzyl piperazines	BZP(*N*-benzylpiperazine)<br>2C-B-BZP<br>MDBP/MDBZP (1-(3,4-methylenedioxybenzyl)piperazine)- methylene-deoxy analogue of BZP, Piperonylpiperazine<br>FBZP (1-(4-fluorobenzyl)-piperazine)<br>MBZP (1-(4-methylbenzyl)-piperazine)<br>DBZP (1,4-dibenzylpiperazina)
Phenyl piperazines	mCPP (1-(3-chlorophenyl)piperazine)<br>pCPP 1-(4-Chlorophenyl)piperazine)<br>mCPCPP (1-(3-Chlorophenyl)-4-(3-chloropropyl)piperazine)<br>TFMPP (1-(3-trifluoromethylphenyl)piperazine)<br>1- TFMPP<br>2- TFMPP<br>3- TFMPP<br>4- TFMPP<br>MePP (1-methyl-3-phenylpiperazine)<br>MeBP (1-(3-methylbenzyl)piperazine)<br>pMeOPP (1-(4- methoxyphenyl)piperazine)<br>oMeOPP (1-(2-Methoxyphenyl)piperazine)<br>pFPP (1-(4-fluorophenyl)-piperazine)<br>DCPP (2,3-dichlorophenylpiperazine)<br>mMPP (1-(3-Methylphenyl)piperazine)<br>pMPP (1-(4-Methylphenyl)piperazine)

Table S2. Intoxications with piperazines.

Age/sex	Quantity ingested	Compound detected in the toxicological screening	Sample	Reference
16, Female	4 tablets	BZP	n.a	[1]
17, Male	5 tablets	n.a	Serum, urine	[2]
18, Female	n.a	BZP	n.a	[1]
18, Female	4 tablets	BZP (260–270 ng/mL), TFMPP (30–60 ng/mL)	Serum	[3]
18, Female	4 tablets			
19, Male	4 tablets			
19, Female	n.a	BZP (0.20 mg/L) and metabolites	Plasma, urine, blood	[4]
20, Male	3 – 4 tablets	BZP	Blood	[5]
22, Male	3 – 4 tablets	BZP (2.23 mg/L), MDMA (1.05 mg/L)	Plasma	[4]
23, Female	n.a	BZP	Serum, plasma	[6]
25, Male	4 tablets	BZP	n.a	[1]
29, Female	3 tablets	mCPP (320 ng/mL)	Plasma	[7]
		mCPP (2300 ng/mL)	Urine	
88 patients, 15 – 42 years	aproximately 3.89 tablets	BZP	Plasma	[8]
7 patients, 18 – 23 years	3 – 9 tablets	BZP (1.3, 1.9, 1.9, and 2.5 mg/L)	Serum	[9]

n.a.: not available

Table S3. Examples of natural and synthetic tryptamines

TRYPTAMINES

**Commum Name**	**Abbreviation**	**Molecular formula**	**Chemical Structure**
		**Natural Origin Tryptamines**	
Dimethyltryptamine	DMT	$C_{12}H_{16}N_2$	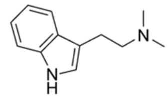
Psilocybin		$C_{12}H_{17}N_2O_4P$	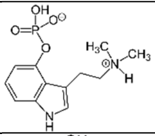
Psilocin	4-OH-DMT	$C_{12}H_{16}N_2O$	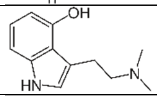
5-Methoxy-*N,N*-dimethyltryptamine	5-MeO-DMT	$C_{13}H_{18}N_2O$	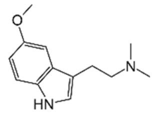
5-Hydroxy-*N,N*-dimethyltryptamine	Bufotenine	$C_{12}H_{16}N_2O$	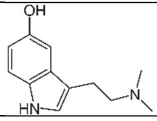
		**Tryptamines of Synthetic Origin**	
α-methyltryptamine	*α*-MT	$C_{11}H_{14}N_2$	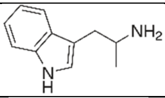
α-ethyltryptamine	*α* -ET	$C_{12}H_{16}N_2$	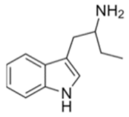
N,N-diallyltryptamine	DALT	$C_{16}H_{19}N_2$	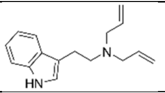
Dipropyltryptamine	DPT	$C_{16}H_{24}N_2$	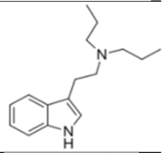
N-methyltryptamine	NMT	$C_{11}H_{14}N_2$	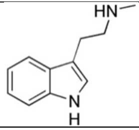
Diisopropyltryptamine	DiPT	$C_{16}H_{24}N_2$	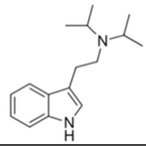
Diethyltryptamine	DETECTIVE	$C_{14}H_{20}N_2$	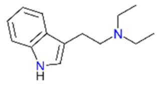

N-methyl-*N* ethyltryptamine	MET	$C_{13}H_{18}N_2$	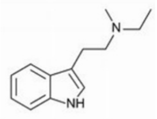
4-acetoxy-*N*,*N*-diethiltryptamine	4-AcO-DET	$C_{16}H_{22}N_2O_2$	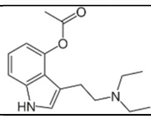
4-Acetoxy-*N*-methyl-*N*-ethyltryptamine	4-AcO-MET	$C_{15}H_{20}N_2O_2$	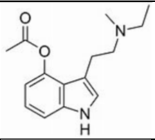
4-Acetoxy-*N*,*N*-dipropyltryptamine	4-AcO-DPT	$C_{18}H_{26}N_2O_2$	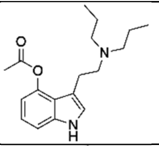
4-Acetyloxy-*N*,*N*-diallyltryptamine	4-AcO-DALT	$C_{18}H_{22}N_2O_2$	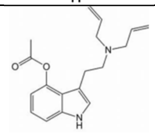
4- acetoxi-*N*,*N*-diisopropyltryptamine	4-AcO-DiPT	$C_{18}H_{26}N_2O_2$	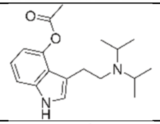
4-Hydroxy-*N*-methyl-*N*-ethyltryptamine	4-OH-MET	$C_{13}H_{18}N_2O$	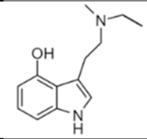
4-hydroxy-*N*,*N*-diisopropyltryptamine	4-OH-DiPT	$C_{16}H_{24}N_2O$	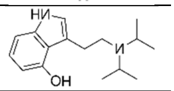
4-Hydroxy-*N*,*N*-dipropyltryptamine	4-OH-DPT	$C_{16}H_{24}N_2O$	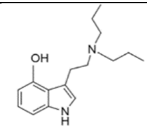
4-hydroxy-*N*,*N*-diethyltryptamine	4-OH-DET	$C_{14}H_{20}N_2O$	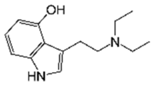
4-hydroxy-*N*-methyl-*N*-isopiltryptaline	4-OH-MiPT	$C_{14}H_{20}N_2O$	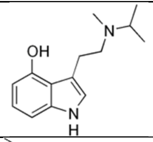
5-Methoxy-*N*,*N*-diallyltryptamine	5-MeO-DALT	$C_{17}H_{22}N_2O$	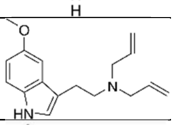
5-Methoxy-*N*,*N*-diethyltryptamine	5-MeO-DET	$C_{15}H_{22}N_2O$	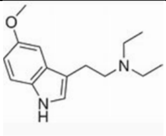
5-Methoxy-α-methyltryptamine	5-MeO-α-MT	$C_{12}H_{16}N_2O$	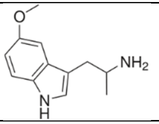

5-Methoxy-*N*-methyl-*N*-ethyltryptamine	5-MeO-MET	$C_{14}H_{20}N_2O$	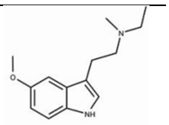
5-Methoxy-*N*,*N*-diisopropyltryptamine	5-MeO-DiPT "Foxy Methoxy"	$C_{17}H_{26}N_2O$	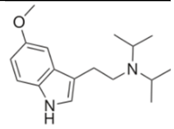
5-Methoxy-*N*,*N*-dipropyltryptamine	5-MeO-DPT	$C_{17}H_{26}N_2O$	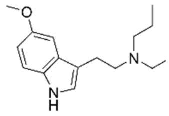
5-methoxy-*N*,*N*-dimethiltryptamine	5-MeO-DMT	$C_{13}H_{18}N_2O$	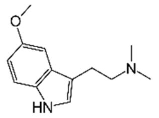
5-methoxy-*N*-methyl-*N*-isopiltryptaline	5-MeO-MiPT "Moxy"	$C_{15}H_{22}N_2O$	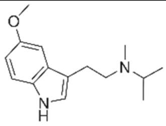
5-Methoxy-*N*,*N*-diethyltryptamine	5-MeO-DET	$C_{15}H_{22}N_2$	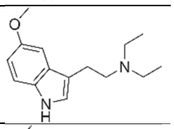
5-Methoxy-*N*,*N*-trimethyltryptamine	5-MeO-TMT	$C_{14}H_{20}N_2O$	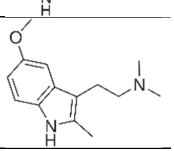
	**Ergolines**		
(8β)-9,10-Didehydro-6-methyl-ergoline-8-carboxamide	LSA	$C_{16}H_{17}N_3O$	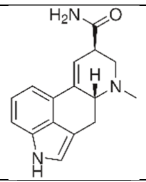
9,10-didehydro-*N*,*N*-diethyl-6-methylergoline-8β-carboxamide	LSD	$C_{20}H_{25}N_3O$	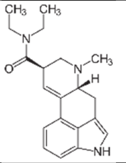

Table S4. Classes of SCRA and their chemical characterization

Category	Structure	Examples of compounds
Classical cannabinoids	Similar to THC	HU-210; HU211; AM-906; AM-411
Non-classical cannabinoids	Cyclohexylphenols	CP compounds: CP-47,497 and analogues;
Hybrid cannabinoids	Share similarities with classical and non-classical cannabinoids	AM-4030
Aminoalkylindoles	Assimilar to THC	WIN 55,212-2; AM-1241, UR-144, 5F-APICA; compounds of the JWH family
Eicosanoids	Structures analogous to endocannabinoids	methanandamide
Others	Other structures; Diapyrazoles, naphtaylpyrroles and naphthylmethylindenes	SR141716A; SR144528

Table S5. Phencyclidine-type substances

Abbreviation (common name)	Chemical name
Ketamine	2-(2-Chlorophenyl)-2-(methylamino)cyclohexan-1-one
PCA	1-Phenylcyclohexan-1-amine
PCP (phencyclidine)	1-(1-phencyclohexyl) piperidine
PCE (eticyclidine)	*N*-ethyl-1-phenylcyclohexylamine
PCPr	*N*-Propyl-1-phenylcyclohexylamine
PCiP	1-Phenyl-*N*-(propan-2-yl)cyclohexan-1-amine
PCPy, PHP (rolicyclidine)	1-(1-phenylcyclohexyl) pyrrolidine
PCMo	1-(1-Phenylcyclohexyl)morpholine
TCP (tenocyclidine)	1-[1-(thiophen-2-yl)cyclohexyl]piperidine
TCPy	1-[1-(Thiophen-2-yl)cyclohexyl]pyrrolidine
2-MeO-PCP	2-methoxy-phencyclidine
3-OH-PCP (3-hydroxyphencyclidine	3-[1-(Piperidin-1-yl)cyclohexyl]phenol
3-OH-PCE	3-[1-(Ethylamino)cyclohexyl]phenol
3-MeO-PCE (methoxieticyclidine)	2-(3-methoxyphenyl)-2-(ethylamino)cyclohexane
3-MeO-PCP (3-methoxyphencyclidine)	1-[1-(3-methoxyphenyl)cyclohexyl]piperidine
3-MeO-PCPy (3-methoxyrolicyclidine)	1-[1-(3-Methoxyphenyl)cyclohexyl]-pyrrolidine
3-MeO-PCPr	2-(3-Methoxyphenyl)-2-(propylamino)cyclohexane
4-Me-PCP	1-[1-(4-Methylphenyl)cyclohexyl]piperidine
4′-Me-PCP	4-Methyl-1-(1-phenylcyclohexyl)piperidine
4-MeO-PCP (methoxydine)	1-[1-(4-methoxyphenyl)cyclohexyl]piperidine
5-MeO-PCP (5-methophencyclidine)	1-[1-(5-methoxyphenyl)cyclohexyl]piperidine
MXE (methoxetamine)	2-(3-Methoxyphenyl)-2-(ethylamino)cyclohexan- 1-one

Table S6. Case report intoxications with phencyclidine-type substances.

Age/sex	Via of administration	Case report	Detected compound	Quantity	Symptoms	Reference
126 patients, median age of 22 years	>50% nasally and oral	Non-fatal	Mostly just Ketamine, but in other cases co-ingested alcohol (10.3 %), ecstasy (6.4 %), and methamphetamine (6.0 %).	Not mentioned	The most common symptoms were hypertension and tachycardia. Other symptoms were nausea or vomiting, dysuria, abdominal tenderness, abnormal LFTs, dilatation of the CBD, cystitis, chronic abdominal pain and psychiatric concerns.	[10]
17, male	Nasally	Non-fatal	MXE	Not mentioned	Reduced level of consciousness, severe truncal ataxia, dysarthria, dysdiadochokinesis, incoordination and horizontal nystagmus.	[11]
45, male	Oral	Non-fatal	4-MeO-PCP and ethanol	Not mentioned	Disorientation, hypersalivation, tremors and occasional myoclonic jerks, scanning speech with dysarthria, and nystagmus in all directions of lateral gaze	[12]
54, male	Oral	Fatal	4-MeO-PCP and 4-HO-MET	Blood- 8,200 ng/mL<br>Urine-140 mg/L<br>Gastric contents-280 mg	Not mencioned	[12]
17, male	1º time:<br>Oral 200 mg<br><br>2º time:<br>Nasally 50mg	Non-fatal	3-MeO-PCP	1º time:<br>Blood-71.1 ng/mL<br>Urine-706 ng/L<br><br>2ºtime:<br>Not mentioned	Hypertension, tachycardia and neurological manifestations such as confusion, hypertonia, nystagmus and agitation	[13]
29, male	Not mencioned	Fatal	3-MeO-PCP	139 ng/mL of 3-MeO-PCP, with 4.1 mg/L of	Congested lungs and distended bladder	[12]

	diphenhydramine and 100 ng/mL of amphetamine	

References

1. Gee, P.; Richardson, S.; Woltersdorf, W.; Moore, G. Toxic effects of BZP-based herbal party pills in humans: A prospective study in Christchurch, New Zealand. *N. Z. Med. J.* **2005**, *118*, 1–10.
2. Alansari, M.; Hamilton, D. Nephrotoxicity of BZP-based herbal party pills: a New Zealand case report. *N. Z. Med. J.* **2006**, *119*, 1–3.
3. Wood, D.M.; Button, J.; Lidder, S.; Ramsey, J.; Holt, D.W.; Dargan, P.I. Dissociative and sympathomimetic toxicity associated with recreational use of 1-(3-trifluoromethylphenyl) piperazine (TFMPP) and 1-benzylpiperzine (BZP). *J. Med. Toxicol.* **2008**, *4*, 254–257.
4. Gee, P.; Jerram, T.; Bowie, D. Multiorgan failure from 1-benzylpiperazine ingestion legal high or lethal high. *Clin. Toxicol.* **2010**, *48*, 230–233.
5. Austin, H.; Monasterio, E. Acute psychosis following ingestion of 'Rapture.' *Australas. Psychiatry* **2015**, *12*, 406–408.
6. Balmelli, C.; Kupferschmidt, H.; Rentsch, K.; Schneemann, M. Tödliches hirnödem nach einnahme von ecstasy und benzylpiperazin. *Dtsch. Medizinische Wochenschrift* **2001**, *126*, 809–811.
7. Kovaleva, J.; Ir, E.D.; Paepe, P. De; Verstraete, A. Acute chlorophenylpiperazine overdose: A case report and review of the literature. *Ther. Drug Monit.* **2008**, *30*, 394–398.
8. Gee, P.; Gilbert, M.; Richardson, S.; Moore, G.; Paterson, S.; Graham, P. Toxicity from the recreational use of 1-benzylpiperazine. *Clin. Toxicol.* **2008**, *46*, 802–807.
9. Elliott, S. Current awareness of piperazines: pharmacology and toxicology. *Drug Test. Anal.* **2011**, *3*, 430–438.
10. Ho, J.H.; Dargan, P.I. Arylcyclohexamines (Ketamine, Phencyclidine, and Analogues). In *Critical Care Toxicology*; Springer International Publishing: Cham, 2016; pp. 1–46.
11. Shields, J.E.; Dargan, P.I.; Wood, D.M.; Puchnarewicz, M.; Davies, S.; Waring, W.S. Methoxetamine associated reversible cerebellar toxicity: Three cases with analytical confirmation. *Clin. Toxicol.* **2012**, *50*, 438–440.
12. Wallach, J.; Brandt, S.D. Phencyclidine-based new psychoactive substances. In *Handbook of Experimental Pharmacology*; Springer, Cham, 2018; Vol. 252, pp. 261–303.
13. Berar, A.; Allain, J.-S.; Allard, S.; Lefevre, C.; Baert, A.; Morel, I.; Bouvet, R.; Gicquel, T. Intoxication with 3-MeO-PCP alone. *Medicine (Baltimore).* **2019**, *98*, e18295.
